# Reducing Annotation Costs Using Knowledge Distillation for Label‑Efficient Negation Detection in Radiology Reports: Multisite Internal Validation Study

**DOI:** 10.2196/103912

**Published:** 2026-10-22

**Authors:** Kung-Hsun Weng, Bo-Lin Huang, Yu-Ting Kuo, Yi-Chen Chou, Tsyh-Jyi Hsieh, Chung-Feng Liu

**Affiliations:** 1 Department of Medical Imaging Chi Mei Medical Center Tainan Taiwan; 2 Institute of Precision Medicine College of Medicine National Sun Yat-sen University Kaohsiung Taiwan; 3 Department of Radiology School and College of Medicine Kaohsiung Medical University Kaohsiung Taiwan

**Keywords:** radiology report, natural language processing, negation, deep learning, transfer learning, supervised learning, validation study, transformers, BERT, knowledge distillation, clinical application, radiology

## Abstract

**Background:**

Negation in radiology reports (eg, “no evidence of dissection”) is essential for accurately conveying diagnostic information. Clinical natural language processing systems, however, often fail to determine whether findings occur within negated or speculative contexts, leading to excessive false keyword alerts, noise in information extraction, and reduced precision in downstream clinical or research applications. Transformer models have advanced detection capabilities but require costly expert labeling. Large language models (LLMs) may provide label-efficient alternatives through few-shot prompting or knowledge distillation.

**Objective:**

This study aims to systematically evaluate LLMs and transformer models under different supervision strategies, including comparisons of expert-labeled and LLM-distilled transformers, teacher LLMs (GPT-5.3 Chat, Gemma 4, and Qwen3.6-Plus), sampling approaches, and direct few-shot LLM inference, to determine whether LLM inference or LLM-based distillation can substitute for human labeling in radiology-report negation detection while reducing annotation costs.

**Methods:**

We reused and analyzed 6000 deidentified English-language radiology reports from 3 hospital branches. Three LLMs—GPT-5.3 Chat, Gemma 4, and Qwen3.6-Plus—performed 0-, 5-, and 10-shot inference to identify negations and speculations unrelated to abnormal findings and generate pseudo-labels for transformer fine-tuning (Bio_ClinicalBERT [Bio + Clinical BERT model], BERT [Bidirectional Encoder Representations from Transformers], and ALBERT [A Lite Bidirectional Encoder Representations from Transformers]). We trained models using human-labeled or LLM-distilled supervision across 28 training data scales (n=42-4200) and 3 data sampling seeds and compared them across multiple supervision and training conditions encompassing backbone, label source, and sampling strategy. We assessed performance using keyword-level precision, recall, and F1-score with paired bootstrap tests (10,000 replicates).

**Results:**

We refined 1000 report annotations (interrater κ=0.957) and fine-tuned 1260 transformer models. Human-labeled transformers consistently outperformed distillation-labeled ones, with the largest gap at small training scales (ΔF1-score ≈0.042; adjusted *P*<.001), which narrowed at larger scales (ΔF1-score ≈0.022; adjusted *P*<.001). Teacher model choice had minimal effect on distilled performance. All 3 human-labeled transformer backbones (BERT, ALBERT, and Bio_ClinicalBERT) achieved high keyword-level F1-scores (0.982-0.985 under their best conditions; n=4200). In the 10-shot setting, GPT-5.3 Chat (F1-score 0.974) modestly underperformed human-labeled transformers in the large-sample range (ΔF1-score=0.009; adjusted *P*=.047) and significantly outperformed Gemma 4 (F1-score 0.957; adjusted *P*=.01) and Qwen3.6-Plus (F1-score 0.950; adjusted *P*<.001) in the bootstrap analysis. GPT-5.3 Chat labeled reports at approximately US $0.018 per report and became less expensive than manual annotation when expert hourly wages exceeded US $0.64-1.50.

**Conclusions:**

Human-supervised transformers remain the strongest baseline, but frontier LLMs such as GPT-5.3 Chat offer comparable performance with minimal supervision. LLM-based distillation offers a cost-effective alternative with only minor performance loss. Dataset scale proved more influential than sampling strategy. Additionally, superior teacher performance yielded no proportional gains through distillation, as open-weight or low-cost teachers achieved comparable results. If supported by external validation, the proposed label-efficient framework could enable accurate and affordable negation detection and rapid domain adaptation.

## Introduction

### Background

Negation in radiology reports (eg, “No evidence of aortic dissection”) is crucial for excluding critical conditions and conveying diagnostic information. However, natural language processing (NLP) systems still struggle to determine whether medical concepts occur in negated or speculative contexts that do not indicate abnormal findings. Such errors may lead to downstream consequences that adversely affect health care, such as redundant keyword alerts and reduced clinician trust—the “cry-wolf” effect—when up to 40% of highlighted terms are false positives [1]. Ignoring negation also diminishes the precision of information extraction [2], leading to additional manual review and errors in downstream clinical, research, and educational tasks.

Transformer-based models have markedly advanced negation detection [1,3,4], but their performance depends on large, expert-annotated corpora and may decline under domain shifts [5] as reporting styles change. By contrast, large language models (LLMs) demonstrate strong semantic understanding and can achieve competitive results through few-shot learning without full retraining [6]. LLMs may also serve as teacher models for knowledge distillation (KD) [7], reducing annotation costs and enhancing scalability. This study therefore investigates frontier LLMs and label-efficient supervision strategies that leverage LLMs to support reliable clinical NLP systems for radiology report negation detection.

### Review of Literature

#### Historical Approaches to Negation and Speculation Detection

Negation and speculation detection in clinical text has progressed from rule-based algorithms [8-13] to modern deep learning–based approaches [1,3,4,14-21]. Early rule-based tools [8-13] used lexical patterns, with or without syntactic parsing, to define negation scope but struggled with text containing unusual grammar or requiring cross-sentence analysis. Machine learning methods [22-26] learned statistical cues from labeled corpora and outperformed rule-based models, yet remained constrained by handcrafted features and annotation demands.

With deep learning [14-17,19-21], convolutional neural networks learned salient local context, whereas recurrent neural network–based models (eg, long short-term memory and gated recurrent unit models) better captured sequential dependencies and broader semantic relationships. Recent studies [1,3,4,18] show that fine-tuned transformers achieve strong performance in negation detection; however, their performance may depend on large human-labeled datasets. For instance, our previous work [1] used 6000 annotated radiology reports, including 4500 training samples.

#### KD and Label Efficiency in Clinical NLP

KD transfers the predictive behavior of a large “teacher” model to a smaller “student” model, enabling compact architectures to retain accuracy while reducing computational requirements [27,28]. Widely adopted in NLP, KD enables smaller transformers to approximate larger models by mimicking their output distributions or internal representations. For example, DistilBERT (Distilled version of Bidirectional Encoder Representations from Transformers) reduces BERT’s (Bidirectional Encoder Representations from Transformers) number of parameters by approximately 40% while retaining most of its linguistic capabilities [29].

The rise of LLMs extends KD into a new paradigm in which powerful models such as GPT-3 generate high-quality pseudo-labels without task-specific fine-tuning [7]. Transformers trained on these labels can approach human-labeled baselines while reducing annotation costs by up to 50%-96% [7], although LLM-derived labels remain less reliable than expert annotations [30].

Recent studies have introduced KD into clinical NLP, including the compression of clinical transformers [28] and the enhancement of information extraction from biomedical text [31,32] and public health communication [33]. However, none of these studies has systematically evaluated KD as a label-efficient supervision strategy for radiology report negation detection or directly compared few-shot LLMs with human-labeled transformers for this specific task.

### Study Aim

This study systematically evaluated LLMs and transformers under multiple supervision strategies to detect negation and speculation unrelated to abnormal findings in radiology reports across different training data scales. We investigated the following:

- Whether KD from LLMs can substitute for expert labeling;
- How teacher model choice affects student performance;
- How sampling strategy influences overall F1-score and recall for negation subtypes;
- How student transformers compare with their LLM teachers;
- How human-labeled transformers compare with few-shot LLMs; and
- How different LLMs—including open-weight and state-of-the-art black-box models—perform under a fixed few-shot setting.

By analyzing multiple supervision conditions and data scales, we aimed to determine when direct LLM deployment, distillation-based transformer fine-tuning, or human-labeled fine-tuning is most effective and to provide practical guidance for implementing and maintaining negation detection systems in health care settings.

## Methods

### Ethical Considerations

The Chi Mei Hospital Institutional Review Board (approval number 11410-020) approved the study. We deidentified all data for this retrospective analysis, obtained a waiver of informed consent, and provided no compensation to participants.

### Study Design

Figure 1 illustrates the study workflow. The first author (KHW) analyzed 6000 labeled radiology reports from our previous dataset [1]. The second author (BLH) independently annotated 1000 samples to assess interrater agreement, after which both authors resolved discrepancies. Multiple LLMs and transformer-based models processed the reports to identify negations and speculations unrelated to abnormal findings. We compared their keyword-level performance against human-annotated gold-standard labels.

**Figure 1:**
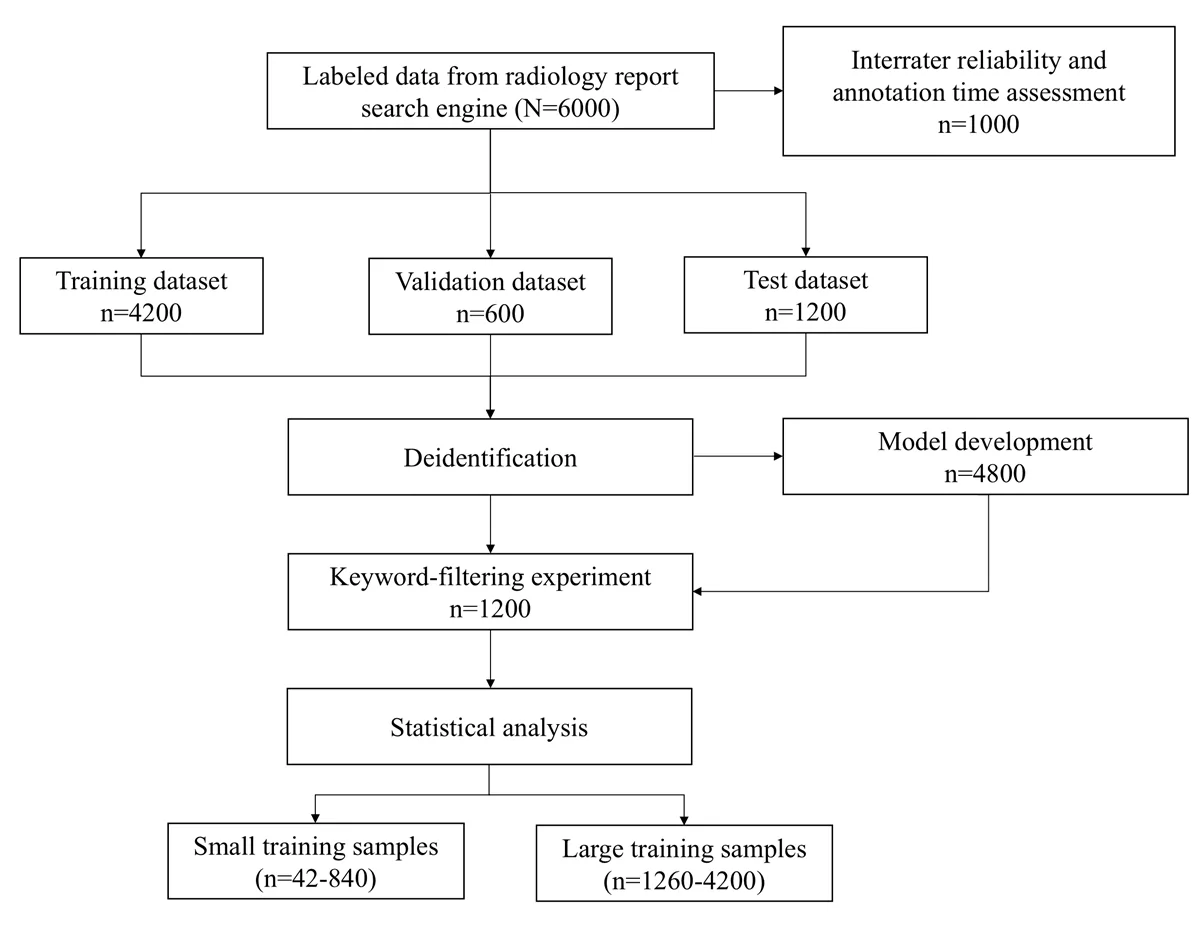
Study workflow.

### Inclusion and Exclusion Criteria

Our reused dataset comprised English-language X-ray, computed tomography (CT), magnetic resonance imaging (MRI), ultrasound, and special radiology examinations collected between 2012 and 2022 from the 3 hospital branches. We excluded Chinese-language reports and patients younger than 20 years, consistent with the applicable legal age of majority during the data collection period [34]. We compiled the dataset using the following keyword searches: bleeding, consolidation, dissection, extravasation, fracture, infarct, metastasis, pneumoperitoneum, pneumothorax, pulmonary TB, thrombosis, and tumor. We derived these keywords from the institution’s predefined list of alert terms that trigger highlighting or pager notifications in radiology reports.

### Dataset Preparation

#### Deidentification

We deidentified all included radiology reports by removing identifying information, including medical record numbers, application numbers, examination dates, ordering departments, and examination times.

#### Interrater Agreement Assessment

We inherited the dataset from our previous study, in which the primary annotator (KHW) generated the original labels. For this study, we formalized the previous annotation guidelines without substantively changing the annotation criteria to enable independent annotation by a second annotator.

After finalizing the annotation guidelines, we randomly selected 1000 reports from our dataset to assess interrater agreement using Cohen κ.

Author BLH, a physician coauthor in postgraduate training, independently annotated the reports, while the primary annotator (KHW) reannotated the same cases. KHW and BLH calculated word-level agreement, resolved discrepancies by consensus, and revised the labels accordingly.

#### Human and Computational Cost Estimation

##### Overview of Break-Even Cost Analysis

We performed an illustrative break-even analysis comparing manual expert annotation with LLM-assisted pseudo-label generation for the full training dataset (n=4200). The LLM-assisted workflow included prompt engineering, GPT-5.3 Chat inference, and 2-pass dynamic programming (DP)–based text alignment. For manual annotation, we extrapolated the total annotation time from a small subset of expert-annotated samples, considering scenarios with and without familiarity-related efficiency gains. We defined the break-even hourly wage as the expert wage at which the 2 approaches incurred equal estimated costs. As annotation efficiency gains, prompt-engineering effort, API pricing, and computational infrastructure vary across settings, we considered these estimates context-specific rather than universal. We report transformer fine-tuning costs separately because both workflows required fine-tuning, leaving the break-even threshold unchanged.

##### Annotation and Prompt Engineering Time Estimation

The primary annotator (KHW) optimized the prompts for LLM inference. As this time was not recorded prospectively, we retrospectively estimated the total prompt engineering effort from experimental logs by multiplying the number of optimization iterations by the estimated time per iteration. The primary annotator estimated the average duration of each iteration.

The second annotator (BLH) recorded the labeling time, which we extrapolated to 4200 reports to estimate the total time, assuming an efficiency gain of up to 100% with task familiarity [35]. We also conducted a sensitivity analysis assuming no familiarity-related efficiency gains.

##### Computational Cost Estimation for Transformer Models and Text Alignment

We rented a cloud instance from Vast.ai (Intel Core i5-14400F CPU, 32 GB RAM, NVIDIA RTX 5080 GPU, and 2.5 TB SSD [solid-state drive]) at US $0.633 per hour. We estimated the computational costs of 3 processes by multiplying the execution time of each process by the hourly rental rate: training ALBERT (A Lite Bidirectional Encoder Representations from Transformers) on the largest human-labeled training dataset (n=4200 reports), running ALBERT inference on the full test dataset (n=1200 reports), and performing 2-pass DP text alignment on the 10-shot GPT-5.3 Chat inference results for the full test dataset (n=1200 reports).

##### GPT-5.3 Chat Inference Cost Estimation

We recorded the input, cached input, and output tokens consumed by GPT-5.3 Chat on the test dataset. We calculated inference costs by multiplying the numbers of uncached input tokens (total input tokens minus cached input tokens), cached input tokens, and output tokens by their respective per-token prices at the time of the study (US $1.75, US $0.18, and US $14.00 per million tokens, respectively) and summing the resulting costs. We then calculated the mean cost per report and extrapolated the total cost to the full training dataset (n=4200 reports).

We performed LLM inference, excluding 2-pass DP text alignment, on the authors’ existing local, consumer-grade PC rather than the rented Vast.ai instance, thereby avoiding additional Vast.ai rental costs.

#### Data Annotation

We refined the labels for negations and speculations unrelated to abnormal findings using our in-house software, which served as the gold standard for analysis. Annotators labeled spans throughout the entire report rather than only the predefined target keywords. We labeled each token as either category 0 (positive or speculative statements potentially related to abnormal findings) or category 1 (negative or speculative statements unrelated to abnormal findings), as in our previous study (Table 1) [1].

**Table 1:** Classification of words and tokens in our study.

Type and subtypes		Example^a^	Token classification^b^
**Negative**			
	Sentential negation	Tibial shaft fractures *without displacement*.	1
	Symbolic or telegraphic-style negation	*(–) Metastasis* *Dense MCA sign: No*	1
	Morphological negation	*Nontumoral lesion*	1
**Speculative**			
	Speculation unrelated to abnormal findings	*No CT evidence of acute infarct. Suggest MRI to exclude hyperacute infarct if indicated.*	1
	Speculation potentially related to abnormal findings	Rt parietal lobe acute infarct cannot be excluded. R/O pneumonia	0
**Positive**			
		Rt parietal lobe acute infarct.	0

^a^The token class indicates the italicized text only. All nonitalicized text is assigned to category 0.

^b^Category 0 represents positive statements or speculations potentially associated with abnormal findings, and category 1 represents negation or speculations unrelated to abnormal findings.

As in our previous work, we designed the annotation scheme to optimize downstream clinical workflows, including clinical alerts, keyword highlighting, and case retrieval, rather than general-purpose negation detection. Accordingly, we used a task-specific annotation scheme.

For our downstream task, explicit negations and speculations unrelated to abnormal findings both indicate the absence of disease presence or suspicion in radiology reports. We therefore annotated them as a single category to align with the downstream clinical objective.

For secondary analysis, we further divided category 1 tokens into 4 linguistic patterns but treated them as a single class during model evaluation. Following our previous work [1], we categorized negations as sentential, symbolic, or morphological. We identified speculation unrelated to abnormal findings in 2 situations: (1) when the radiologist explicitly denied a finding, for example, “No evidence of A; suggest exam B to rule out A if indicated,” or (2) when a diagnosis could not be evaluated or excluded because of study limitations, such as motion artifacts, technical issues, or suboptimal contrast.

#### Dataset Partition

We reshuffled the dataset and split it into training, validation, and test sets at a ratio of 7:1:2 (4200, 600, and 1200 reports, respectively). The validation set was used solely for early stopping during transformer fine-tuning.

We split the test dataset without enforcing patient-level separation, allowing follow-up studies from the same individuals to appear across subsets. This design reflects real-world practice, in which new data naturally include patients who undergo multiple examinations.

We also constructed an engineering dataset comprising approximately 80 training samples for iterative LLM prompt refinement.

### Model Development

#### Overview of the Model Development and KD Framework

Figure 2 summarizes the model development and KD framework.

**Figure 2:**
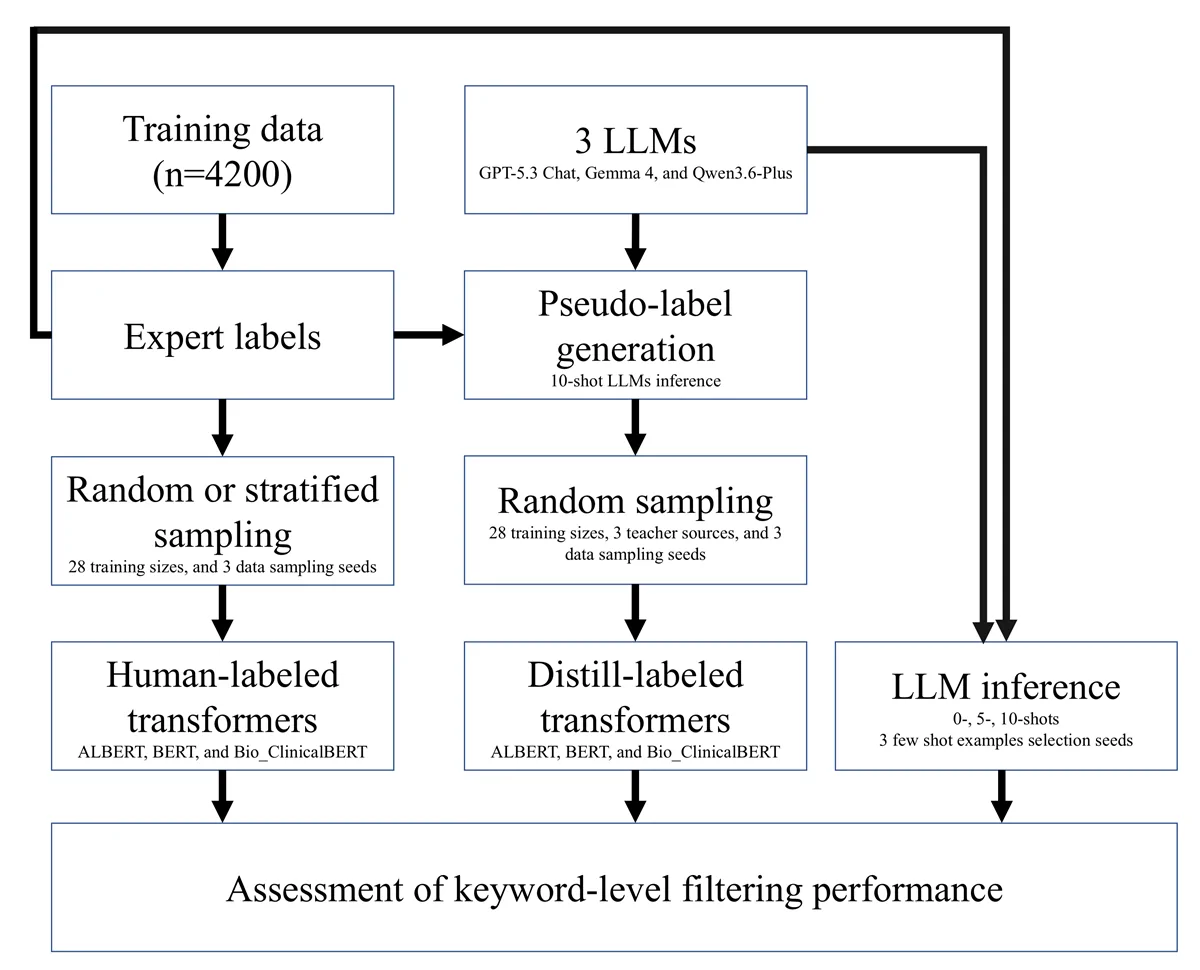
Model-development and knowledge-distillation framework. ALBERT: A Lite Bidirectional Encoder Representations from Transformers; BERT: Bidirectional Encoder Representations from Transformers; Bio_ClinicalBERT: Bio + Clinical BERT model; LLM: large language model.

We fine-tuned 3 transformer backbones across 28 training data sizes and 3 data-sampling seeds, using expert labels with random or stratified sampling or pseudo-labels generated by 3 LLMs through 10-shot prompting with random sampling. We also evaluated the LLMs through direct 0-, 5-, and 10-shot inference, using 3 example-selection seeds for the 5- and 10-shot settings. Figure 3 illustrates the keyword-level evaluation process for all models.

**Figure 3:**
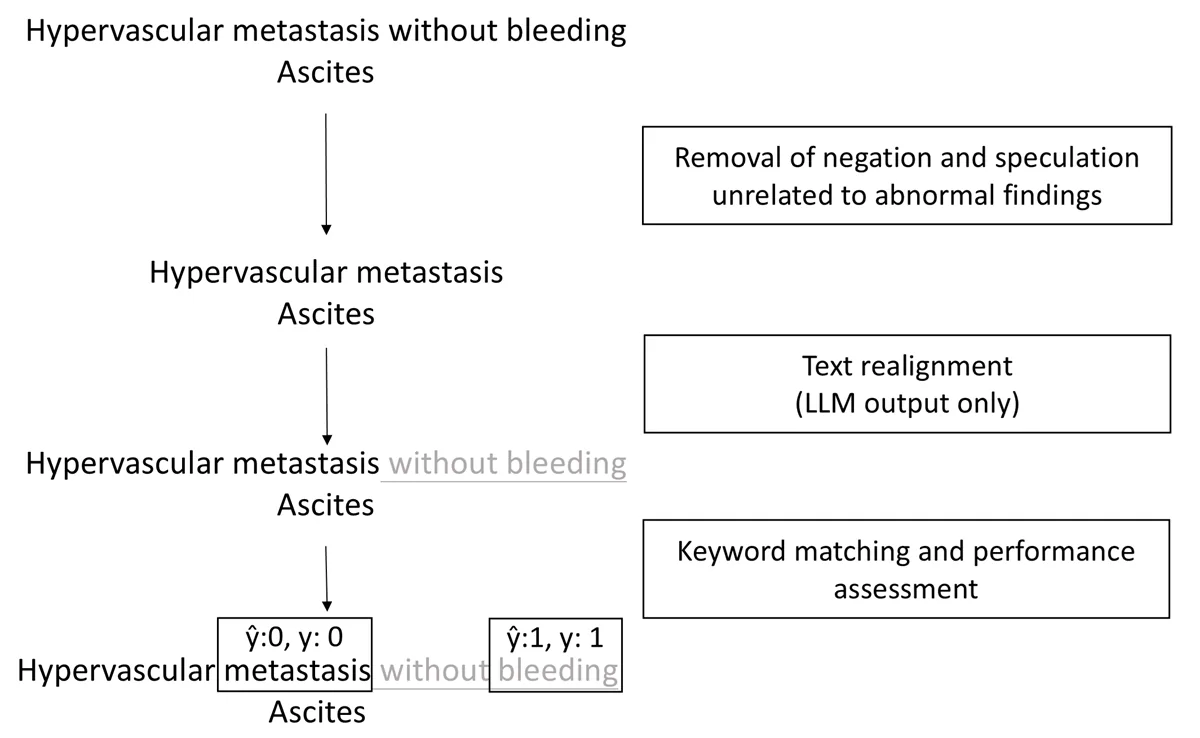
Model evaluation process. We replaced each underlined segment with white space of equal length to keep text alignment. The system predicted matched keywords in filtered text as class 0 and white space–replaced keywords as class 1. We compared predicted classes (ŷ) with gold labels (y) to compute word‑level performance metrics. LLM: large language model.

#### Large Language Models

##### Prompt Development and Optimization

We designed a structured, rule-guided prompt (Multimedia Appendix 1) to instruct LLMs to remove negations and speculations unrelated to abnormal findings while keeping all other content unchanged. A board-certified radiologist iteratively optimized the prompt using GPT-5.3 Chat and the engineering dataset, adjusting errors until zero-shot performance plateaued. We abstracted common linguistic patterns and avoided reproducing original sentences. The final prompt contained no test-specific patterns or phrases.

The prompt defined 4 mutually exclusive negation and speculation categories (Table 1), with representative example phrases, and directed the LLM to provide reasoning for each removal, including the negation type, trigger words, justification, and confidence (0-1). If the LLM detected no negation, it returned the original text unchanged with an empty reasoning array.

For sensitivity analysis, we also performed lightweight, model-specific prompt adaptation using the engineering dataset to evaluate its impact on LLM performance. We incorporated brief clarifications describing the linguistic patterns most frequently missed by each model into the original GPT-5.3 Chat–optimized prompt while preserving its overall structure. The adapted prompts were used only for this analysis and not to generate pseudo-labels for subsequent KD experiments or to conduct few-shot experiments on the full test dataset.

##### Stratified Few-Shot Sample Selection

We constructed few-shot samples from the training dataset and grouped all reports into 4 predefined negation subtypes (Table 1) to balance minority patterns. For a given number of shots (N), we evenly distributed examples across categories—for example, 3, 3, 2, and 2 reports for n=10. Reports could belong to multiple groups if they contained multiple negation subtypes. Within each group, we shuffled reports using 3 fixed random seeds (1000, 1357, and 5566) and selected unique samples sequentially without duplication to maintain balanced representation, reproducibility, and sample uniqueness.

##### Model Selection and Implementation

We evaluated 3 LLMs using a standardized, API-based inference pipeline: GPT-5.3 Chat (OpenAI), Qwen3.6-Plus (Alibaba), and the open-weight Gemma 4 31B Instruct (Google).

We accessed GPT-5.3 Chat through the OpenAI API and the other 2 models through OpenRouter (Stripe, Inc). We applied uniform prompting, few-shot sampling, and output parsing across models, setting the temperature to 0 and the random seed to 5566 when supported to ensure consistent, deterministic inference.

##### Word‑Level Alignment of Filtered Texts

We aligned LLM outputs with the original text using a 2-pass DP algorithm to enable accurate performance evaluation and generate distilled labels for transformer training. The first pass calculated the minimum edit distance, and the second minimized word-boundary breaks among optimal edit paths to avoid fragmenting words.

During backtracking, the algorithm discarded text inserted by the LLM because it had no corresponding character positions in the original report, whereas deleted source text was replaced with white space at the corresponding positions. Consequently, keyword-level evaluation used only the filtered original report and was therefore unaffected by text inserted by the LLM. As the algorithm solved a global edit-distance optimization problem and deterministically selected 1 optimal alignment in the second pass, it always produced at least one valid alignment, even when the LLM removed every source character and generated entirely hallucinated text; in such cases, the deleted source positions were represented as white space.

This process established a consistent character-position mapping between the raw and filtered texts. For example, aligning “You candy” with “You cannot eat candy” produces “You (space) candy” rather than “You can (space) dy,” ensuring that the word “candy” remains contiguous. Our planned hospital information system implementation uses algorithmically filtered text rather than raw LLM output.

#### Transformer Models

##### Token-Character Mapping

We implemented a tokenizer-independent training and inference pipeline. During both stages, we used the tokenizer’s offset mapping to associate each subword token with its corresponding character span. During fine-tuning, we assigned class 1 to subword tokens whose character spans overlapped with labeled spans and class 0 to all other tokens. During inference, we projected predicted class 1 subword tokens back to character-level masks using the same offset mapping. We then performed keyword matching and evaluation using predicted and gold character–level masks rather than tokenizer-specific subword sequences, ensuring consistent evaluation across different transformer tokenizers.

##### Model Selection and Implementation

We fine-tuned transformer models using the Hugging Face (Hugging Face, Inc) framework with 3 fixed random seeds (1000, 1357, and 5566) to ensure reproducibility. For each seed, the training subset was resampled according to the assigned sampling strategy, and the model was fine-tuned separately on each sampled dataset.

Each model processed sequences of up to 512 tokens with a batch size of 8 and was trained for up to 60 epochs (learning rate 2.0 × 10^–5^, weight decay 0.01, and linear warmup 0.1), with early stopping after 3 validation rounds. We did not perform hyperparameter searches; instead, we used identical hyperparameters across all transformer backbone models to ensure fair comparisons among backbone models and supervision strategies.

We fine-tuned 3 pretrained models: Bio_ClinicalBERT (Bio + Clinical BERT model) (emilyalsentzer/Bio_ClinicalBERT), BERT-Large-Uncased (bert-large-uncased), and ALBERT-XXLarge-v2 (albert-xxlarge-v2). For long reports, we segmented the text into chunks of up to 384 tokens while preserving sentence boundaries.

##### Multifactor Model Training Design

We systematically investigated transformer-based filtering of radiology reports using a controlled multifactorial design, examining how training size, model architecture, label source, sampling strategy, and data sampling seeds influenced performance.

We trained ALBERT, BERT, and Bio_ClinicalBERT across data scales ranging from 1% (42/4200) to 20% (840/4200), in 1% (42/4200) increments, and from 30% (1260/4200) to 100% (4200/4200), in 10% (420/4200) increments. Labels came from expert annotations or distillations from GPT-5.3 Chat, Gemma 4, and Qwen3.6-Plus using 10-shot prompting. Sampling was either stratified or random; stratified sampling was used only for human-labeled data because LLM-predicted negation subtypes lacked human verification. Each fine-tuned transformer model represented a unique combination of backbone, data size, label source, sampling strategy, and data seed.

#### Assessment of Keyword-Level Filtering Performance

As shown in Figure 3, our experiment followed a structured 3-stage workflow to assess keyword-level filtering performance.

First, we removed negations and speculations unrelated to abnormal findings using both LLMs and transformers to process all test-set reports. Each model aimed to detect and remove tokens labeled as class 1 by experts.

Second, we performed text realignment. After filtering, we realigned each LLM output with the original report using the aforementioned 2-pass DP algorithm.

Third, we performed keyword matching and evaluation. Although annotators labeled spans throughout each report, we evaluated performance at the predefined keyword level because this evaluation reflects the intended clinical workflows, such as keyword highlighting and case retrieval. For each target high-priority keyword (bleeding, consolidation, dissection, extravasation, fracture, infarct, metastasis, pneumoperitoneum, pneumothorax, pulmonary TB, thrombosis, and tumor) [1], we determined, based on the prefiltered text, whether it occurred within a negated or speculative context unrelated to abnormal findings (y=1) and whether the model masked it (ŷ=1). We then computed keyword-level precision, recall, and *F*_1_-scores and derived subtype-specific recall across 4 negation subtypes to assess model performance. All performance metrics in our study reflect keyword-level filtering performance for predefined clinical alert keywords, not general span-level negation detection, unless otherwise specified.

### Statistical Analysis

#### Overview of the Analytical Framework

This study included 2 complementary analytical frameworks. Primary and secondary comparative analyses (comparison families A-G) used 10,000 paired bootstrap resamples of held-out test reports to estimate paired differences in *F*_1_-scores and, for subtype-specific analyses, recall, along with 95% CIs and multiplicity-adjusted *P* values.

Descriptive performance and sensitivity analyses were conducted directly on the original test dataset without bootstrap resampling. These analyses included performance under maximal-data conditions, sensitivity analyses of patient overlap, and variability across data sampling seeds.

#### Bootstrapped Paired Evaluation

We performed all statistical analyses in Python 3.13.5 (Python Foundation). We set the significance level to α=.05 and used 2-sided tests unless stated otherwise.

Using a paired bootstrap framework, we compared the primary outcome (keyword-based *F*_1_-score) and the secondary outcome (recall for each of the 4 negation subtypes).

We generated 10,000 bootstrap replicates by sampling with replacement from the 1200-report test dataset and computed group performance on the same set of replicates to ensure strict pairing across all comparison families.

For each contrast between groups A and B within a fixed training-size range, we defined the paired difference as Δr = metric_A,r_ − metric_B,r_. The metrics for groups A and B (metric_A,r_ and metric_B,r_, respectively) were averaged across all compatible seed-specific model configurations, including training data size, backbone architecture, label source, sampling strategy, and data seeds, as applicable.

The empirical distribution of Δr yielded the mean difference, the 95% percentile CI, and the 2-sided empirical *P* value: 2 × min(P[Δr≥0], P[Δr≤0]). We controlled for multiplicity using the Holm-Bonferroni procedure and rejected the null hypothesis (Δ=0) when the adjusted *P* value was <α. The 95% CIs were not adjusted for multiplicity.

#### Definition of Comparison Families

##### Overview

We organized the statistical comparisons into 7 families across predefined training-size ranges. Each family assessed specific contrasts in model performance.

##### Comparison Family A: Human-Labeled Versus Distill-Labeled Transformers

We compared mean *F*_1_-scores between transformers trained on human-labeled data and those trained on LLM-distilled labels, both using random sampling.

##### Comparison Family B: Distill-Labeled Transformers by Teacher Source

We performed pairwise contrasts among transformers trained on labels distilled from GPT-5.3 Chat, Qwen3.6-Plus, and Gemma 4 to examine the influence of the teacher LLM on student performance.

##### Comparison Family C: Sampling Strategy Under Human Labeling

We compared the mean *F*_1_-scores of human-labeled transformers trained using stratified and random sampling.

##### Comparison Family D: Student Group Versus Corresponding Teacher

We compared each distilled transformer with its corresponding teacher LLM.

##### Comparison Family E: Human-Labeled Versus Each LLM

We compared human-labeled transformers trained using both stratified and random sampling, with each LLM evaluated under a fixed 10-shot setting.

##### Comparison Family F: Among LLMs

We conducted pairwise comparisons among the LLMs (GPT-5.3 Chat, Qwen3.6-Plus, and Gemma 4) to identify differences in performance.

##### Comparison Family G: Per-Category Recall Under Human Labeling

We compared mean recall across all negation subtypes between stratified and random sampling to assess subtype-specific recall.

#### Definition of Training‑Sample Size Ranges

In this study, we considered training sample sizes below 1000 to represent relatively small-scale transformer training [36]. We selected 840 as the cutoff, as it is close to 1000 and corresponds to 20% (840/4200) of the full training set, to distinguish between small- and large-sample conditions.

For analyses A, B, C, D, E, and G, primary inferences were made across the small-sample (n=42-840) and large-sample (n=1260-4200) ranges, followed by secondary exploratory analyses at each training size.

#### Post Hoc Error-Pattern Analysis Under Best-Case Pseudo-Label Supervision

We conducted a post hoc descriptive analysis of residual limitations at the case level under best-case pseudo-label supervision. We selected the 10-shot LLM with the highest *F*_1_-score as the teacher and, at the maximum training set size, identified the transformer architecture with the smallest *F*_1_-score reduction under pseudo-label supervision relative to its human-supervised counterpart. Both models used the same backbone, 4200 training reports, and sampling strategy. We then manually reviewed test cases that the human-supervised transformer classified correctly but the pseudo-label–supervised transformer misclassified, along with the corresponding predictions from the LLM teacher.

#### Sensitivity Analysis for Patient Overlap

We conducted a descriptive sensitivity analysis by excluding patients in the test dataset who also appeared in the training or validation sets and recalculating *F*_1_-scores for models evaluated under maximal-data conditions. For each model configuration, *F*_1_-scores were averaged across 3 data sampling seeds before and after exclusion, and the corresponding difference (Δ*F*_1_-score) was reported. We did not perform bootstrap resampling or hypothesis testing for this analysis.

#### Sensitivity Analysis of Data Seed Variability

We conducted a descriptive sensitivity analysis of multiseed variability on the original test dataset without bootstrap resampling. We evaluated 5- and 10-shot LLM inference across 3 few-shot example selection seeds and all transformer configurations across 3 training-data sampling seeds used during fine-tuning. For each configuration, we calculated keyword-level *F*_1_-scores for each seed and reported the mean and sample SD of the 3 seed-specific *F*_1_-scores (Multimedia Appendix 2).

## Results

### Demographics

Following our previous study [1], we used a dataset of 6000 radiology reports authored by 78 residents and attending physicians in the radiology department. The dataset included 2538 plain radiography reports, 2163 CT reports, 668 MRI reports, 483 ultrasound reports, 97 angiography reports, and 51 reports from other modalities.

The corpus comprised 78,901 sentences and 704,512 words. After refining the annotations, we labeled 105,140 (14.92%) words, corresponding to 19,465 span-based labels, as negative or speculative statements unrelated to abnormal findings. We defined a “word” as the longest contiguous sequence of non–white space characters; for example, “(–) hypertension” =2 words. Of these labels, 16,362 (84.06%) were classified as sentential, 2770 (14.23%) as symbolic, 135 (0.69%) as morphological negations, and 198 (1.02%) as speculations unrelated to abnormal findings (Table 1).

Table 2 summarizes the distribution of the 12 target keyword occurrences across the training, validation, and test datasets. All target keywords appeared in every dataset partition, although their frequencies varied. We used the same version of spaCy [37] and the same keyword-counting procedure as in our previous study [1] to calculate keyword occurrence frequencies for descriptive characterization of the dataset.

**Table 2:** Distribution of target keyword occurrences across the training, validation, and test datasets. Values represent the total occurrences and frequencies of each target keyword in each dataset partition. Repeated keywords within a report were counted separately because performance was evaluated at the individual keyword-occurrence level.

Keywords	Training (n=8017), n (%)	Validation (n=1107), n (%)	Test (n=2385), n (%)
Bleeding	92 (1.15)	19 (1.72)	6 (0.25)
Consolidation	316 (3.94)	38 (3.43)	74 (3.10)
Dissection	477 (5.95)	54 (4.88)	141 (5.91)
Extravasation	149 (1.86)	12 (1.08)	21 (0.88)
Fracture	1554 (19.38)	216 (19.51)	410 (17.19)
Infarct	970 (12.10)	126 (11.38)	268 (11.24)
Metastasis	1275 (15.90)	162 (14.63)	438 (18.36)
Pneumoperitoneum	44 (0.55)	7 (0.63)	12 (0.50)
Pneumothorax	902 (11.25)	142 (12.83)	263 (11.03)
Pulmonary TB	9 (0.11)	4 (0.36)	2 (0.08)
Thrombosis	416 (5.19)	65 (5.87)	137 (5.74)
Tumor	1813 (22.61)	262 (23.67)	613 (25.70)

### Interrater Agreement Assessment

We randomly sampled 1000 radiology reports (116,405 words) for independent annotation, yielding an interannotator agreement of κ=0.957.

### Human and Computational Cost Estimation

Table 3 summarizes the estimated human labor and computational costs of the evaluated workflows.

**Table 3:** Estimated human labor and computational costs of the evaluated workflows.

Tasks	Dataset	Effort/cost	Basis	Assumption
Prompt engineering	Engineering^a^	6-12 hours	6 optimization iterations	1-2 hours per iteration
Manual labeling	Training^b^	63-126 hours	3 hours for 100 reports	100% efficiency gain with task familiarity
GPT-5.3 Chat inference	Training	US $76.4	N/A^c^	Extrapolated^d^
GPT-5.3 Chat inference	Test^e^	US $21.8	7,864,252 input; 30,016 cached input; and 580,468 output tokens (35 minutes and 11 seconds)	N/A
Two-pass dynamic programming	Training	US $0.31	N/A	Extrapolated
Two-pass dynamic programming	Test	US $0.09	498.5 seconds	N/A
ALBERT^f^ fine-tuning	Training	US $0.37	2,091.3 seconds	N/A
ALBERT inference	Test	US $0.01	57.0 seconds	N/A

^a^The engineering dataset (about 80 reports).

^b^The maximal training dataset (n=4200 reports).

^c^N/A: not applicable.

^d^Extrapolated values were estimated from the test dataset.

^e^The test dataset (n=1200 reports).

^f^ALBERT: A Lite Bidirectional Encoder Representations from Transformers.

Based on the costs included in this analysis, GPT-5.3 Chat–assisted annotation—which required 6-12 hours of expert work for prompt engineering, US $76.40 for GPT-5.3 Chat inference on the training dataset, and US $0.31 for 2-pass DP on the training dataset—became less expensive than manual annotation (63-126 hours of expert work) when expert hourly wages exceeded US $0.64-1.50.

After model training, ALBERT inference on the full test dataset cost only US $0.01, compared with US $21.80 for GPT-5.3 Chat.

### Model Performance Under Maximal Data Conditions

To provide an intuitive performance benchmark, we summarized keyword-level *F*_1_-scores and subtype-specific recall under maximal training conditions (n=4200 for transformers and 10-shot prompting for LLMs) in Table 4.

**Table:** Model performance under maximal data conditions with sensitivity analysis in the test dataset, with transformers trained on the full training set (n=4200) and large language models evaluated using the 10‑shot setting.^a^

Model	Label source	Sampling	*F*_1_-score (full)^b^	*F*_1_-score (excluded)^c^	Δ*F*_1_-score^d^	Recall (S)^e^	Recall (T)^f^	Recall (P)^g^	Recall (M)^h^	
Gemma 4	Human	Stratified	0.957	0.963	0.006	0.974	0.815	1.000	N/A^i^	
GPT-5.3 Chat	Human	Stratified	0.974	0.973	–0.001	0.988	0.972	0.926	N/A	
Qwen3.6-Plus	Human	Stratified	0.950	0.955	0.005	0.949	0.937	0.926	N/A	
**BERT^j^**										
	Human	Stratified	0.981	0.981	0.000	0.993	0.949	0.889	N/A	
	Human	Random	0.985	0.985	0.000	0.995	0.968	0.889	N/A	
	Gemma 4	Random	0.962	0.960	–0.002	0.994	0.870	0.963	N/A	
	GPT-5.3 Chat	Random	0.949	0.951	0.002	0.990	0.661	0.519	N/A	
	Qwen3.6-Plus	Random	0.924	0.928	0.004	0.977	0.690	0.778	N/A	
**ALBERT^k^**										
	Human	Stratified	0.982	0.982	0.000	0.992	0.968	0.778	N/A	
	Human	Random	0.977	0.978	0.000	0.993	0.951	0.667	N/A	
	Gemma 4	Random	0.970	0.970	0.000	0.987	0.931	0.963	N/A	
	GPT-5.3 Chat	Random	0.972	0.973	0.001	0.989	0.937	0.778	N/A	
	Qwen3.6-Plus	Random	0.947	0.946	–0.001	0.943	0.945	0.889	N/A	
**Bio_ClinicalBERT^l^**										
	Human	Stratified	0.984	0.985	0.001	0.993	0.968	1.000	N/A	
	Human	Random	0.984	0.985	0.001	0.993	0.968	1.000	N/A	
	Gemma 4	Random	0.963	0.963	–0.001	0.994	0.907	0.815	N/A	
	GPT-5.3 Chat	Random	0.969	0.968	0.000	0.992	0.919	0.704	N/A	
	Qwen3.6-Plus	Random	0.967	0.965	–0.002	0.990	0.931	0.815	N/A	

^a^*F*_1_-scores and subtype-specific recalls were averaged across 3 data seeds. For direct large language model inference, “Label Source” and “Sampling” indicate the source and sampling strategy for few-shot exemplars, respectively, and the 3 data seeds correspond to different selections of few-shot exemplars. We did not fine-tune large language models.

^b^Performance on the full test dataset.

^c^Performance on the test dataset after excluding patients overlapping with the training and validation dataset.

^d^Performance difference: *F*_1_-score (excluded) – *F*_1_-score (full).

^e^Recall (S): Recall of sentential negation (n=982).

^f^Recall (T): Recall of symbolic or telegraphic-style negation (n=169).

^g^Recall (P): Recall of speculation unrelated to abnormal findings (n=9).

^h^Recall (M): Recall of morphological negation; keyword absent in the test dataset (n=0), precluding calculation.

^i^N/A: not applicable.

^j^BERT: Bidirectional Encoder Representations from Transformers.

^k^ALBERT: A Lite Bidirectional Encoder Representations from Transformers.

^l^Bio_ClinicalBERT: Bio + Clinical BERT model.

All 3 human-labeled transformer backbones (BERT, ALBERT, and Bio_ClinicalBERT) achieved comparably high keyword-level *F*_1_-scores (0.982-0.985 under their best sampling conditions). Transformers trained on distilled labels performed slightly below their human-labeled counterparts. For LLMs in the 10-shot setting, GPT-5.3 Chat scored highest (*F*_1_-score 0.974), followed by Gemma 4 (*F*_1_-score 0.957) and Qwen3.6-Plus (*F*_1_-score 0.950). These results provide a practical benchmark and complement the formal bootstrap-based comparative analyses presented below.

For subtype-specific recall, sentential-negation recall remained high (0.943-0.995) across configurations, whereas recall of symbolic or telegraphic-style negation varied by backbone and label source. Relative to human-labeled models trained using random sampling, recall decreased by 0.098-0.307 for BERT, 0.006-0.020 for ALBERT, and 0.037-0.061 for Bio_ClinicalBERT across different pseudo-label sources, with ALBERT showing the smallest decrease in recall.

Recall of speculation unrelated to abnormal findings ranged from 0.519 to 1.000 but should be interpreted with caution, as only 9 related keywords were present in the test dataset. No morphological-negation keywords were present in the test dataset, precluding calculation of recall. Subtype-specific precision and *F*_1_-scores were unavailable because false positives in transformer output lacked subtype assignments.

### Post Hoc Error-Pattern Analysis Under Best-Case Pseudo-Label Supervision

We selected ALBERT because replacing expert labels with GPT-5.3 Chat–generated pseudo-labels produced the smallest reduction in mean *F*_1_-scores across multiple seeds under maximal-data random sampling (n=4200 reports; 0.977 vs 0.972). For feasibility, we selected the paired human-supervised and distilled runs corresponding to data sampling seed 5566 without comparing the seed-specific performance or error profiles of the 3 paired runs.

Among 29 keyword occurrences across 22 test reports that the human-supervised ALBERT classified correctly but the distilled student misclassified, 13 (45%) were teacher-concordant errors, in which the teacher and student made the same incorrect classification. The remaining student-specific errors comprised 11 (38%) false positives in which only the student incorrectly filtered the keyword, 4 (14%) student false negatives involving symbolic or telegraphic-style negation, and 1 (3%) student false negative involving sentential negation.

### Sensitivity Analyses

#### Overview of Sensitivity Analysis

Table 5 summarizes 4 sensitivity analyses addressing assumed gains in annotation efficiency, patient overlap, model-specific prompt adaptation, and random seed selection. The following subsections report the corresponding quantitative results and methodological details.

**Table 5:** Summary of major sensitivity analyses.

Methodological concern	Sensitivity analysis	Effect on study findings
Assumption of annotation efficiency gain	No familiarity-related improvement in manual labeling efficiency	Break-even cost conclusion remained similar
Patient overlap	Exclusion of test-set patients overlapping with the training or validation sets	Negligible effect on performance estimates
Model-specific prompt sensitivity	Lightweight model-specific prompt adaptation	Minor and inconsistent changes in large language model performance
Dependence on specific seed selection	Evaluation across 3 data or few-shot example-selection seeds	Generally limited variability, with greater instability at smaller training sizes

#### Sensitivity Analysis of the Annotation Efficiency Assumption

Assuming no efficiency gain from labeling familiarity, GPT-5.3 Chat–assisted labeling (requiring 6-12 hours of prompt engineering) became less expensive than 126 hours of expert annotation at hourly wages above US $0.64-0.67.

#### Sensitivity Analysis to Patient Overlap

We evaluated potential patient-level data leakage by reassessing the Table 4 models after removing 253 out of 1111 (22.77% in the test dataset) unique patients who overlapped with the training and validation datasets. Δ*F*_1_-scores ranged from –0.002 to 0.006, with mixed directions, consistent with random variation and suggesting a negligible impact of patient overlap on performance evaluation.

#### Sensitivity Analysis of Prompt Adaptation

The lightweight adaptation yielded only minor and inconsistent changes in performance (*F*_1_-scores: Gemma 4, 0.878-0.858; Qwen3.6-Plus, 0.836-0.858). This analysis used only the engineering dataset (about 80 reports) under the zero-shot setting rather than 10-shot performance on the held-out test dataset. We developed and evaluated the adapted prompts using the same engineering dataset; therefore, the evaluation was not independent of prompt development.

#### Sensitivity Analysis of Data Seed Variability

We evaluated 1260 transformer seed-specific configurations, comprising 420 base configurations defined by training set size, backbone, label source, and sampling strategy, each evaluated using 3 training data sampling seeds.

Keyword-level *F*_1_-scores showed low but nonnegligible variability (Multimedia Appendix 2). On the full test set, the median SD was 0.012 (IQR 0.005-0.022; range 0.000-0.077). Small-sample configurations (n=42-840; 300 base configurations) showed greater variability (median SD 0.015; IQR 0.008-0.025) than large-sample configurations (n=1260-4200; 120 base configurations; median SD 0.006; IQR 0.003-0.012).

Most base configurations showed limited seed-related variability: 295 of 420 (70.2%) base configurations had an SD ≤0.020, calculated from unrounded values. However, a minority, predominantly those with smaller training sizes, showed greater variability. The largest SD occurred for ALBERT trained on Qwen3.6-Plus–distilled labels with n=252 under random sampling (mean *F*_1_-score 0.897; SD 0.077). Overall, transformer performance remained generally stable across seeds, although some configurations showed substantial seed-related variability, especially in the small-sample regime.

Across the 3 evaluated LLMs, observed SDs were numerically higher with 5-shot prompting (median 0.010; range 0.003-0.021) than with 10-shot prompting (median 0.007; range 0.006-0.008).

### Primary Comparative Analysis

Table 6 summarizes the primary comparisons across the small-sample (n=42-840) and large-sample (n=1260-4200) ranges (see per-N results in Multimedia Appendix 3).

**Table 6:** Mean differences (ΔF1-score or ΔRecall) with 95% bootstrap CIs^a^.

Families^b^		Small samples (n=42-840)		Large samples (n=1260-4200)	
		Mean difference (95% CI)	*P* value	Mean difference (95% CI)	*P* value
**A. Human^c^—distill^d^**		0.042 (0.011 to 0.076)	*<.001*	0.022 (0.011 to 0.031)	*<.001*
	Gemma 4	0.045 (0.001 to 0.076)	*.02*	0.021 (0.012 to 0.040)	*<.001*
	GPT-5.3 Chat	0.041 (0.009 to 0.083)	*<.001*	0.016 (0.006 to 0.027)	*<.001*
	Qwen3.6-Plus	0.040 (0.018 to 0.077)	*<.001*	0.029 (0.012 to 0.044)	*<.001*
**B. Among distill**					
	GPT-5.3 Chat-Gemma 4	0.004 (–0.039 to 0.028)	>.99	0.005 (–0.004 to 0.016)	.88
	GPT-5.3 Chat-Qwen3.6-Plus	–0.001 (–0.026 to 0.026)	>.99	0.013 (–0.002 to 0.028)	.47
	Qwen3.6-Plus-Gemma 4	0.005 (–0.022 to 0.035)	>.99	–0.008 (–0.023 to 0.016)	.88
*C. Human: stratified sampling—random sampling*		0.011 (–0.005 to 0.028)	.15	–0.002 (–0.007 to 0.002)	.80
**D. Student—teacher**					
	Gemma 4	–0.043 (–0.082 to –0.013)	*<.001*	0.006 (–0.011 to 0.020)	.86
	GPT-5.3 Chat	–0.056 (–0.100 to –0.017)	*<.001*	–0.007 (–0.020 to 0.003)	.74
	Qwen3.6-Plus	–0.032 (–0.081 to 0.009)	.22	0.005 (–0.010 to 0.021)	.86
**E. Human—large language model**					
	Human-Gemma 4	0.007 (–0.044 to 0.028)	.91	0.026 (0.014 to 0.040)	*<.001*
	Human-GPT-5.3 Chat	–0.010 (–0.061 to 0.008)	.91	0.009 (0.000 to 0.017)	*.047*
	Human-Qwen3.6-Plus	0.014 (–0.037 to 0.035)	.91	0.033 (0.020 to 0.046)	*<.001*
**F. Among large language models**					
	GPT-5.3 Chat-Qwen3.6-Plus	0.024 (0.014 to 0.034)	*<.001*	0.024 (0.014 to 0.034)	*<.001*
	GPT-5.3 Chat-Gemma 4	0.017 (0.005 to 0.031)	*.01*	0.017 (0.005 to 0.031)	*.01*
	Qwen3.6-Plus-Gemma 4	–0.007 (–0.021 to 0.007)	.34	–0.007 (–0.021 to 0.007)	.34
**G. Recall of negation subtypes**					
	Sentential	–0.002 (–0.013 to 0.002)	>.99	0.000 (–0.003 to 0.001)	>.99
	Telegraphic	0.105 (0.002 to 0.408)	.16	0.003 (–0.017 to 0.023)	>.99
	Pseudo-speculation	0.137 (N/A^e^)	N/A	–0.036 (N/A)	N/A
	Morphological	N/A	N/A	N/A	N/A

^a^Performance was averaged across eligible model configurations (training size, architecture, label source, sampling strategy, and data seeds) and then computed paired differences. A positive Δ*F*_1_-score indicates a higher mean *F*_1_-score for the first setting. We performed 2-sided paired bootstrap tests (10,000 replicates) and controlled multiplicity using the Holm-Bonferroni procedure. Italicized values indicate results were significant at α=.05 after adjustment. The 95% CIs were not adjusted for multiplicity. Families A–F report differences in *F*_1_-score (Δ*F*_1_-score), whereas family G reports differences in subtype-specific recall (ΔRecall).

^b^Comparison families A-G correspond to those defined in the “Statistical Analysis” section. See Multimedia Appendix 2 for the complete list of model configurations. In the test dataset, morphological negation was absent (n=0), precluding calculation of the corresponding statistics. Given the rarity of pseudo-speculation (n=9), we report its point estimates descriptively and recommend cautious interpretation.

^c^Human: Transformers trained with human labels.

^d^Distill: Transformers trained with distilled labels.

^e^N/A: not applicable.

Transformers trained on human annotations outperformed those trained on distilled labels, with a larger performance gap at small sample sizes. In post hoc analyses, all 3 sources showed statistically significant differences under both small-sample conditions (Gemma 4, adjusted *P*=.02; GPT-5.3 Chat, adjusted *P*<.001; Qwen3.6-Plus, adjusted *P*<.001) and large-sample conditions (all 3 sources, adjusted *P*<.001).

Performance did not differ significantly among distilled students from different teachers. By contrast, GPT-5.3 Chat outperformed Gemma 4 (Δ*F*_1_-score 0.017, 95% CI 0.005-0.031; adjusted *P*=.01) and Qwen3.6-Plus (Δ*F*_1_-score 0.024, 95% CI 0.014-0.034; adjusted *P*<.001). Stratified sampling did not improve *F*_1_-score or recall for any negation subtype.

GPT-5.3 Chat–distilled (Δ*F*_1_-score –0.056, 95% CI –0.100 to –0.017; adjusted *P*<.001) and Gemma 4–distilled (Δ*F*_1_-score –0.043, 95% CI –0.082 to –0.013; adjusted *P*<.001) transformers underperformed their teacher models at small sample sizes but converged at larger scales.

When compared with LLMs, human-labeled transformers slightly but significantly outperformed GPT-5.3 Chat in the large-sample range (Δ*F*_1_-score 0.009, 95% CI 0.000-0.017; adjusted *P*=.047). However, the statistical significance was borderline, warranting cautious interpretation. By contrast, the advantages over Gemma 4 (Δ*F*_1_-score 0.026, 95% CI 0.014-0.040; adjusted *P*<.001) and Qwen3.6-Plus (Δ*F*_1_-score 0.033, 95% CI 0.020-0.046; adjusted *P*<.001) were more pronounced. No significant differences were observed under small-sample conditions (adjusted *P*=.91 for all comparisons).

### Secondary Analysis

#### Comparison Family A: Human-Labeled Versus Distill-Labeled Transformers

Across training sizes, human-labeled transformers outperformed those trained on LLM-distilled labels, with the largest gap at small sample sizes (n=42-840); this gap narrowed but persisted up to n=4200 (see Figure S1A in Multimedia Appendix 4). Post hoc analyses confirmed that all distilled students (GPT-5.3 Chat, Gemma 4, and Qwen3.6-Plus) underperformed their human-labeled counterparts (see Figure S1A, post hoc analyses, in Multimedia Appendix 4), indicating that the performance loss was independent of the teacher model. Overall, KD yielded lower performance than expert supervision, and larger datasets mitigated but did not eliminate the limitations of LLM-generated labels.

#### Comparison Family B: Distill-Labeled Transformers By Teacher Source

Transformers trained on distilled labels from GPT-5.3 Chat, Gemma 4, and Qwen3.6-Plus showed overlapping performance across training sizes (see Figure S1B in Multimedia Appendix 4), with per-N differences fluctuating around zero and no consistent teacher advantage (Multimedia Appendix 3). This pattern mirrored the primary comparative analysis, which found no significant differences among distilled transformers under either small-sample conditions (adjusted *P*>.99 for all 3 pairwise comparisons) or large-sample conditions (GPT-5.3 Chat vs Gemma 4, adjusted *P*=.88; GPT-5.3 Chat vs Qwen3.6-Plus, adjusted *P*=.47; Qwen3.6-Plus vs Gemma 4, adjusted *P*=.88). Although GPT-5.3 Chat performed best in the bootstrap evaluation (comparison family F), its distilled students did not outperform those trained on labels from other teachers, suggesting that interteacher differences were attenuated after distillation.

#### Comparison Family C: Sampling Strategy Under Human Labeling

Stratified and random sampling produced overlapping performance curves across training sizes (see Figure S2C in Multimedia Appendix 4), with per-N differences centered around zero (Multimedia Appendix 3). Stratified sampling showed minor, inconsistent advantages at small training sizes that did not persist across data points. Overall, the sampling strategy had little effect on *F*_1_-scores under expert supervision, whereas training data size appeared to play a more substantial role.

#### Comparison Family D: Student Group Versus Corresponding Teacher

At small training sizes, student models achieved slightly lower *F*_1_-scores than their teachers. As training size increased, the performance gap progressively narrowed, and student models achieved performance comparable to that of their teachers across all 3 LLMs (see Figure S2D in Multimedia Appendix 4 and its zoomed view). Overall, teacher-student differences remained modest and diminished further with larger datasets (Multimedia Appendix 3).

#### Comparison Family E: Human-Labeled Versus Each LLM

Across training sizes, human-labeled transformers showed a steady upward performance curve (see Figure S3 in Multimedia Appendix 4). At small sample sizes, they underperformed GPT-5.3 Chat but quickly surpassed Gemma 4 and Qwen3.6-Plus.

In large samples, they consistently outperformed Gemma 4 and Qwen3.6-Plus and converged toward GPT-5.3 Chat, with closely overlapping curves and small differences. These results agree with the primary comparative analysis, which showed larger performance advantages over Gemma 4 (adjusted *P*<.001) and Qwen3.6-Plus (adjusted *P*<.001) and a small but statistically significant advantage over GPT-5.3 Chat (adjusted *P*=.047) in the large-sample range.

#### Comparison Family F: Among LLMs

As LLMs were evaluated under a fixed 10-shot configuration, their performance was independent of transformer training size (see Figure S3 in Multimedia Appendix 4) and therefore did not warrant per-N secondary analysis.

#### Comparison Family G: Per-Category Recall Under Human Labeling

Sentential negation, the dominant pattern in the test set (n=982), showed almost identical recall curves under stratified and random sampling (see Figure S3 in Multimedia Appendix 4). Morphological negation was absent from the test dataset (n=0), precluding the calculation of recall. Symbolic or telegraphic negation (n=169) varied moderately, without consistent separation between sampling strategies. Speculation unrelated to abnormal findings (n=9) fluctuated substantially, and these small counts warrant caution in interpretation. Overall, stratified sampling did not show a statistically significant recall advantage for sentential negation (adjusted *P*>.99 under both small- and large-sample conditions) or symbolic or telegraphic negation (adjusted *P*=.16 under small-sample conditions and *P*>.99 under large-sample conditions). *P* values were not calculated for pseudo-speculation or morphological negation.

## Discussion

### Principal Findings

We systematically evaluated direct LLM deployment and distillation-based training as scalable alternatives to human-labeled transformers for radiology negation detection across diverse training data scales, supervision sources, model architectures, and sampling strategies. The key findings are summarized below.

### Human Supervision Remains the Strongest Baseline for Transformer Fine‑Tuning

Human-labeled supervision consistently outperformed distillation-based training across all teachers, especially with limited data. ALBERT closely matched larger backbones, supporting prior findings that lightweight models perform well with large-scale expert annotations [1]. High interannotator agreement (κ>0.9) supported the reproducibility of the annotation procedure.

Although increasing the amount of data narrowed the human-distillation gap, it persisted at larger scales, suggesting a systematic bias in LLM-generated labels that scaling alone cannot resolve. Such residual bias may also explain prediction errors in few-shot LLMs, suggesting that LLM-based labeling still lags behind expert supervision in label fidelity and quality.

The sensitivity analysis of data seed variability showed limited seed-related variability across transformer configurations, suggesting that it was unlikely to fully account for the overall performance patterns across training data sizes. However, individual performance estimates, particularly in small-sample settings, warrant cautious interpretation. The patient-overlap sensitivity analysis showed a negligible impact on performance.

### Few-Shot GPT-5.3 Chat Achieved Near Human-Labeled Transformer Performance Without Fine-Tuning

GPT-5.3 Chat showed only a modest performance difference from human-labeled transformers (Δ*F*_1_-score 0.009, 95% CI 0.000-0.017; adjusted *P*=.047; large-sample regime) with 10-shot prompting and no task-specific fine-tuning, suggesting that frontier LLMs offer a practical option when human-annotated data are limited. However, real-world deployment remains cost-constrained: inference cost averaged approximately US $0.018 per report, which could scale substantially for large archives. For example, our institutional archive contained millions of reports. Empirical evidence of continued declines in LLM token prices [38] suggests that few-shot deployment may eventually become economically feasible for large-scale clinical applications.

### Limited Transfer of Teacher Superiority in Distillation

Teacher superiority did not reliably transfer through distillation. Although GPT-5.3 Chat outperformed other LLMs in direct evaluation—and its 10-shot performance was only modestly below that of human-labeled transformers—the distilled students did not outperform those trained on labels from weaker teachers. Even at larger training scales, student models converged to similar performance levels that remained below those of their human-labeled counterparts.

This attenuation of interteacher differences suggests that distillation compresses variability among high-performing teachers. Given that all teacher LLMs achieved strong baseline performance (*F*_1_-score ≥0.950; Table 4), differences in teacher quality were probably too small to affect student outcomes, whereas training data size had a greater influence.

Thus, when candidate teachers already demonstrate high performance, using marginally better but much costlier frontier models may not yield proportional gains. More affordable teachers may serve as viable alternatives if their downstream performance is validated. Even so, frontier models such as GPT-5.3 Chat remain economically competitive, costing less than expert annotation when experts’ hourly wages exceed US $0.64-1.50, based on our reconstructed estimates of labeling and prompt engineering time.

### Limited Observed Impact of Stratified Sampling

Contrary to expectations, the sampling strategy had little effect on *F*_1_-score or subtype-specific recall under expert supervision.

Secondary analysis (comparison family G) showed that common subtypes (sentential and symbolic negation) converged at small training sizes (approximately n=500), indicating early learning and limited room for further improvement. Rare subtypes (speculation unrelated to abnormal findings and morphological negation) were sparse or absent in the test set, limiting their impact on *F*_1_-score regardless of sampling strategy. The absence of significant improvement should not be taken to indicate that stratified sampling is ineffective; rather, the low prevalence of these subtypes and small counts likely reduced statistical power. Future studies may use larger corpora containing more rare instances to clarify this effect.

By contrast, our findings show that at the scale of thousands of samples, overall data volume may influence performance more than subtype-based sampling. In clinical practice, this suggests that expanding randomly sampled datasets may offer greater benefit than labor-intensive early oversampling of specific subtypes. These findings apply to transformer training and do not extend to few-shot LLM prompting.

### Residual Error Patterns Under Best-Case Pseudo-Label Supervision

The controlled ALBERT comparison showed a modest performance reduction when GPT-5.3 Chat–generated pseudo-labels replaced expert labels under otherwise identical conditions, supporting the attribution of the aggregate reduction to the change in supervision source.

Of the 29 selected error occurrences, 13 (45%) were teacher-concordant errors, in which the teacher and student misclassified the same test occurrence. This pattern suggests the possible transfer of systematic teacher error tendencies but cannot establish the propagation of individual errors because the student never encountered the teacher’s test-set predictions during training. The remaining 16 (55%) discrepancies occurred despite correct teacher predictions, consistent with incomplete teacher-student knowledge transfer.

Among the 16 student-specific errors, 11 were false positives, consistent with over-filtering of keywords, and 5 were false negatives; 4 of the 5 false negatives involved symbolic or telegraphic-style negation. Consistent with this pattern, recall for this subtype decreased from 0.951 with human-supervised ALBERT to 0.937 with GPT-5.3 Chat–distilled ALBERT, with larger reductions for some backbones, such as BERT (from 0.968 to 0.661). These findings suggest reduced robustness to compact or atypical negation forms under pseudo-label supervision. Potential contributors may include systematic pseudo-label noise, information loss from hard labels [27], and stochastic fine-tuning variation. Given the scope and methodology of our study, these descriptive findings suggest candidate mechanisms rather than definitively identifying causal mechanisms.

### Generalizability and Cross-Institutional Adaptation

#### Task-Specific Annotation Scheme

We grouped speculative statements unrelated to abnormal findings with explicit negations because both have the same operational consequence in our downstream applications: neither should trigger keyword highlighting or clinical alerts or be returned by a case-finding application designed to identify positive or potentially positive cases. Thus, we defined labels according to the downstream clinical objective rather than conventional linguistic taxonomy.

We do not propose this task-specific scheme as a universal standard; institutions may tailor annotation policies to local workflows. Transformer-based models can accommodate such policies through retraining on locally annotated data, whereas LLM-based approaches can use institution-specific prompts and few-shot examples without retraining. Therefore, our study emphasizes that annotation policies may be tailored to clinical objectives rather than that our scheme should be strictly followed.

#### Interpretation of Keyword-Based Performance Metrics

Our keyword-based metrics may differ from those used for general-purpose negation detection. Although we trained the models on span-based annotations, we primarily evaluated whether they correctly filtered predefined clinical alert keywords. General-purpose evaluations at the character, token, word, or span level may instead penalize discrepancies in negation scope boundaries. Thus, our high keyword-level performance should not be extrapolated to tasks requiring exact scope agreement. For example, the negation spans “No US evidence of thrombosis at bilateral lower extremities” and “No US evidence of thrombosis” both correctly identify the clinically important term “thrombosis” as negated but may receive different token- or word-level scores depending on their agreement with the expert-annotated scope.

#### Institution-Specific Domain Shifts

Even with an identical annotation scheme and evaluation framework, institution-specific differences, such as note types, authors, and linguistic characteristics, may cause domain shifts and degrade model performance [39]. We therefore expect cross-institutional variation in radiology reporting styles, formats, terminology, and radiologist-specific writing patterns to affect model performance.

Institutions may benefit from fine-tuning transformer-based negation detection models on locally annotated data to accommodate differences in annotation schemes and linguistic distributions simultaneously. This need also highlights the practical value of our KD framework, which can efficiently generate large-scale pseudo-labels for training institution-specific models or for subsequent expert refinement, provided that task requirements are similar and modest performance loss is acceptable.

### Comparison to Prior Works

This study presents the first systematic evaluation of few-shot LLMs combined with a KD framework for radiology negation detection. Prior transformer research, including our work [1,4], demonstrated strong performance but did not examine LLM capabilities or address training costs and annotation efficiency.

We extend the application of KD to radiology report negation detection for the first time. Our findings align with prior evidence that LLMs such as GPT-3 can serve as cost-efficient labeling teachers [7] and further extend KD’s application through an alignment-based algorithm that enables token-level prediction with precise entity boundaries and positions—a capability previously recognized as challenging [30]. This fine-grained prediction supports practical clinical applications, including keyword highlighting in laboratory information systems.

We further compared frontier and open-weight LLMs (GPT-5.3 Chat, Gemma 4, and Qwen3.6-Plus) under direct inference and label-distillation conditions. We evaluated KD and data-scale effects to develop a reproducible and clinically feasible framework.

Finally, our work used a large-scale dataset of 6000 real-world radiology reports to assess robustness and scalability.

### Practical Guidelines for Clinical Deployment

#### Clinical Deployment Considerations and Practical Recommendations

While absolute performance is critical, clinical deployment should also consider inference cost and latency, model and API stability, data governance and privacy, output reliability, and system integration.

Fine-tuned transformers provide efficient, predictable output formatting and can be deployed using fixed model versions and local inference on consumer-grade hardware without relying on cloud APIs.

By contrast, proprietary, closed-weight frontier LLMs such as GPT-5.3 Chat offer strong performance but incur API costs and raise cloud-related privacy concerns. Open-weight LLMs, if deployed locally, offer greater control over data and model versions but require substantial infrastructure. However, the evaluated open-weight LLMs underperformed GPT-5.3 Chat in our study. LLM outputs may also require strict validation (eg, 2-pass DP text alignment to ensure accurate character-position mapping to the original unfiltered reports for keyword highlighting) before integration into existing systems.

Based on performance requirements, inference volume, and available annotation and computational resources, we provide practical recommendations for the following deployment scenarios.

#### High-Performance, High-Volume Scenarios

For high-volume negation filtering requiring high performance, human-labeled transformers achieved the highest overall performance, with substantially lower recurring inference costs and latency than direct frontier LLM inference. On 1200 test reports, ALBERT required 57.0 seconds and approximately US $0.01, compared with 35 minutes 11 seconds and approximately US $21.8 for GPT-5.3 Chat.

Thus, with sufficient annotation resources, they remain the most practical option for large-scale institutional deployment.

#### High-Performance, Low-Volume Scenarios

Direct few-shot frontier LLM inference offers a viable alternative for high-performance, low-volume applications, such as filtering CT and MRI impression sections for subsequent retrieval.

With only 10-shot prompting and no task-specific fine-tuning, GPT-5.3 Chat achieved performance close to that of transformers trained on thousands of expert-labeled reports (Δ*F*_1_-score 0.009, 95% CI 0.000-0.017; large-sample regime). Its higher per-report cost may be acceptable at low volumes because it avoids the need to construct large task-specific datasets, fine-tune models, and maintain dedicated models, provided that other institutional requirements are met.

However, our results still favor transformers for high-volume institutional deployment, although declining LLM inference prices over time [38] may broaden the range of workloads for which direct frontier LLM inference becomes economically feasible.

#### Annotation- and Inference Resource–Constrained Scenarios

When both expert-annotation resources and recurring inference budgets are limited, LLM-based KD offers an affordable alternative with a modest performance trade-off. LLM-generated pseudo-labels enable transformer fine-tuning, shifting LLM expenditure from recurring inference to largely one-time annotation costs while retaining the low cost and latency of transformer inference.

Our results showed no consistent performance differences among students trained with different LLM teachers when all teachers achieved strong performance (*F*_1_-score ≥0.950; Table 4). Thus, lower-cost LLMs may serve as practical alternatives to more expensive frontier proprietary models for pseudo-label generation, provided that downstream transformer performance is carefully validated. This strategy may particularly benefit institutions with limited expert-annotation and inference resources.

#### Hybrid Annotation Workflows

Given the strong few-shot performance of frontier LLMs, a hybrid workflow that combines LLM-generated outputs with clinician review can balance scalability and oversight. With high baseline accuracy in LLM outputs, experts need to only revise a limited number of negation spans rather than to annotate everything from scratch, reducing workload while maintaining label quality. Clear guidelines and structured reviews remain critical to avoid anchoring bias and error propagation from LLM predictions.

### Future Work

#### Enabling Token-Level NLP Tasks Through Algorithmic Alignment

We found that integrating a string-alignment algorithm into the distillation pipeline can create word-level correspondence between LLM outputs and source texts, extending model predictions to individual words. Future studies can apply this framework to sequence-labeling or information-extraction tasks, such as named entity recognition [40], where each word’s position may convey important context.

#### Improving Label Quality and Knowledge Transfer

Our analysis of error patterns in models using distilled labels, compared with human-supervised models, identifies 2 targets for improving LLM-based KD.

First, future studies could test causal mechanisms of downstream performance loss by replacing erroneous LLM-generated labels for selected instances, such as those involving symbolic or telegraphic-style negation, with human labels while holding all other labels and experimental factors constant. Performance recovery would support a causal contribution of these errors rather than a mere association.

Second, studies could apply distillation methods with richer teacher information, such as replacing hard pseudo-labels with confidence-aware or probabilistic teacher signals to determine whether richer supervision improves transfer. Such interventions could further distinguish limitations in the teacher’s predictive performance from those introduced by the distillation method.

#### Role of Parameter-Efficient Fine-Tuning

Our study did not employ parameter-efficient fine-tuning (PEFT). PEFT methods, such as LoRA (low-rank adaptation) [41], may reduce the computational and memory requirements of model adaptation, particularly for larger models such as LLMs. However, full fine-tuning of our transformers required modest computational resources, making data annotation a greater resource burden than model training. As PEFT reduces model adaptation costs rather than labeled-data requirements, it complements rather than replaces our KD framework.

Regarding model performance, PEFT may offer additional gains beyond few-shot inference when sufficient task-specific data are available [42]. However, given the high performance of 10-shot GPT-5.3 Chat (*F*_1_-score 0.974), we did not further fine-tune the LLMs using either full-parameter fine-tuning or PEFT. Future studies could assess the benefits of PEFT for larger models.

### Limitations

This study has several limitations. We set temperature=0 to promote deterministic inference, but GPT-5.3 Chat does not expose temperature control via its API, leaving some stochasticity partially uncontrolled. The large test set (n=1200), multiseed few-shot experiment, and 10,000-replicate bootstrap still reduced random effects and stabilized results.

We performed only light prompt adaptation for Qwen3.6-Plus and Gemma 4, rather than exhaustive model-specific optimization. Thus, we cannot rule out the possibility that further prompt engineering may improve individual LLM performance and influence direct LLM comparisons. The direct LLM comparison should be interpreted as a comparison under standardized prompting rather than maximal performance for individual models.

Our bootstrap-based comparative analyses incorporated performance estimates across multiple seeds, reducing the influence of seed-specific fluctuations. Nevertheless, using more seeds could further improve the precision and stability of individual model performance estimates, particularly in small-sample settings. We also did not assess sensitivity to few-shot example ordering.

Budget constraints led to a single deterministic data split, preventing the full removal of data-partition bias. Bootstrapping reduced but did not eliminate this constraint; for example, rare negation subtypes in comparison family G still limited the statistical power of the secondary analysis. The original dataset split also did not enforce patient-level separation, potentially introducing information leakage and affecting performance estimates for unseen patients. However, this setting also reflects an aspect of routine clinical practice, where clinical NLP systems naturally encounter radiology reports from the same patients, particularly during follow-up. Sensitivity analysis also suggested a limited impact.

A single board-certified radiologist performed primary annotations without periodic quality-control review, risking subtle systematic bias, including consistent handling of ambiguous cases, intrarater drift, and fatigue-related undercalling. However, independent reannotation yielded high interrater reliability (κ=0.957), reducing concerns about annotation inconsistency and substantial rater-specific bias, although potential shared systematic bias cannot be completely excluded.

Compared with our previous study [1], a minor discrepancy in keyword occurrence counts remained despite reconstructing the original software environment and using the same version of spaCy. As the reconstructed pipeline reproduced the previously reported keyword counts on the dataset used in our previous study, the discrepancy (55 of 11,509, 0.48%, occurrences) likely reflects subtle differences in automatic text segmentation. As spaCy served only for descriptive dataset characterization, this discrepancy did not affect model development, performance evaluation, or the study conclusions.

We did not perform backbone-specific hyperparameter search for transformer fine-tuning and instead used a shared set of hyperparameters across all backbone models. Nevertheless, the strong performance achieved under the shared hyperparameter setting (eg, the best-performing BERT achieved an *F*_1_-score of 0.985) suggests that this configuration was sufficient for the comparative objective of this study.

We evaluated performance at the predefined keyword level; the reported metrics may not fully characterize general span- or concept-level negation detection performance. In addition, we did not quantify LLM insertion or hallucination rates because they fell outside our keyword-level evaluation, and our planned hospital information system implementation uses algorithmically filtered text rather than raw LLM output. These errors remain distinct considerations when evaluating general free-text generation quality beyond filtering performance. Moreover, keyword occurrences were unevenly distributed in the test dataset, and common keywords contributed disproportionately to the aggregate *F*_1_-score. Thus, the aggregate *F*_1_-score should be interpreted cautiously when assessing performance on rare terms.

Our economic analysis has limitations. We estimated transformer costs using a consumer-grade service rate, whereas clinical deployment may require costlier institutionally approved infrastructure, potentially altering the relative economic advantage. However, our analysis still characterized the cost-saving potential of transformer-based KD, and such a workload is feasible on on-premises consumer-grade hardware. We also retrospectively estimated prompt-engineering time from experimental logs and per-iteration estimates, thereby introducing uncertainty in labor costs.

Lastly, our data originated from 3 branches of a single institution, which may limit linguistic diversity and generalizability across institutions. Without external validation, we cannot quantify performance degradation from cross-institutional domain shifts or assume that our reported performance generalizes to external health care systems.

### Conclusions

Human-supervised transformers remained the strongest baseline for negation detection, while frontier LLMs such as GPT-5.3 Chat achieved only modestly lower performance without task-specific fine-tuning.

Under limited resources, LLM-based distillation provided a cost-effective option with only modest performance loss. Marginal teacher advantages did not benefit student transformers proportionally, as open-weight or low-cost teachers yielded comparable results for distillation. Maintaining large training sets proved more beneficial than early subtype-specific sampling.

If supported by external validation, our framework enables accurate and affordable keyword-based negation detection for smaller institutions and may extend word-level distillation to broader clinical applications and research.

## Data Availability

The source code, including training and evaluation scripts, the string-alignment algorithm, and the GPT-based prompting templates, is publicly available in a GitHub repository [43]. The deidentified radiology report data underlying this study are not publicly available due to institutional privacy regulations but can be obtained from the authors upon reasonable request and institutional approval.

## Appendix

Multimedia Appendix 1 Final GPT-5.3 Chat prompt, output schema, and modified representative few-shot examples.

Multimedia Appendix 2 All transformer base configurations with sensitivity analysis of data seed variability and patient overlap.

Multimedia Appendix 3 Detailed per-N results of secondary analysis of all comparison families (except family F).

Multimedia Appendix 4 Per-N secondary analysis of comparison families A-G.

## Abbreviations

ALBERT: A Lite Bidirectional Encoder Representations from Transformers
BERT: Bidirectional Encoder Representations from Transformers
Bio_ClinicalBERT: Bio + Clinical BERT model
CT: computed tomography
DistilBERT: Distilled version of Bidirectional Encoder Representations from Transformers
DP: dynamic programming
KD: knowledge distillation
LLM: large language model
LoRA: low-rank adaptation
MRI: magnetic resonance imaging
NLP: natural language processing
PEFT: parameter-efficient fine-tuning
SSD: solid-state drive
