# Supplementary material for: Reducing Annotation Costs Using Knowledge Distillation for Label‑Efficient Negation Detection in Radiology Reports: Multisite Internal Validation Study
Source: JMIR Med Inform. 2026 Oct 22;14:e103912. doi: 10.2196/103912 (PMC13740815; doi:10.2196/103912)
Supplement: Supplementary file 2 [file medinform_v14i1e103912_app2.docx]

| Model | N | Label Source | Sampling | Original Test dataset F1, mean (SD) | Overlap-excluded F1, mean (SD) | ΔF1 |
| --- | --- | --- | --- | --- | --- | --- |
| Gemma 4 | 5 | Human | Stratified | 0.936 (0.021) | 0.939 (0.019) | 0.004 |
| GPT-5.3 Chat | 5 | Human | Stratified | 0.974 (0.003) | 0.976 (0.003) | 0.002 |
| Qwen3.6‑Plus | 5 | Human | Stratified | 0.942 (0.010) | 0.945 (0.010) | 0.003 |
| Gemma 4 | 10 | Human | Stratified | 0.957 (0.008) | 0.963 (0.011) | 0.006 |
| GPT-5.3 Chat | 10 | Human | Stratified | 0.974 (0.006) | 0.973 (0.006) | -0.001 |
| Qwen3.6‑Plus | 10 | Human | Stratified | 0.950 (0.007) | 0.955 (0.004) | 0.005 |
| ALBERT | 42 | Human | Stratified | 0.894 (0.041) | 0.892 (0.039) | -0.001 |
| ALBERT | 42 | Human | Random | 0.920 (0.023) | 0.923 (0.023) | 0.002 |
| ALBERT | 42 | Gemma 4 | Random | 0.912 (0.006) | 0.915 (0.007) | 0.003 |
| ALBERT | 42 | GPT-5.3 Chat | Random | 0.911 (0.025) | 0.914 (0.024) | 0.003 |
| ALBERT | 42 | Qwen3.6‑Plus | Random | 0.889 (0.033) | 0.891 (0.030) | 0.002 |
| BERT | 42 | Human | Stratified | 0.913 (0.020) | 0.916 (0.021) | 0.004 |
| BERT | 42 | Human | Random | 0.907 (0.017) | 0.911 (0.018) | 0.005 |
| BERT | 42 | Gemma 4 | Random | 0.912 (0.011) | 0.916 (0.010) | 0.004 |
| BERT | 42 | GPT-5.3 Chat | Random | 0.899 (0.021) | 0.904 (0.022) | 0.005 |
| BERT | 42 | Qwen3.6‑Plus | Random | 0.892 (0.011) | 0.898 (0.009) | 0.005 |
| Bio_ClinicalBERT | 42 | Human | Stratified | 0.926 (0.028) | 0.930 (0.028) | 0.004 |
| Bio_ClinicalBERT | 42 | Human | Random | 0.921 (0.018) | 0.926 (0.018) | 0.005 |
| Bio_ClinicalBERT | 42 | Gemma 4 | Random | 0.920 (0.014) | 0.926 (0.013) | 0.006 |
| Bio_ClinicalBERT | 42 | GPT-5.3 Chat | Random | 0.909 (0.011) | 0.916 (0.012) | 0.007 |
| Bio_ClinicalBERT | 42 | Qwen3.6‑Plus | Random | 0.900 (0.015) | 0.905 (0.015) | 0.005 |
| ALBERT | 84 | Human | Stratified | 0.924 (0.044) | 0.923 (0.046) | -0.001 |
| ALBERT | 84 | Human | Random | 0.920 (0.027) | 0.924 (0.024) | 0.004 |
| ALBERT | 84 | Gemma 4 | Random | 0.882 (0.010) | 0.886 (0.012) | 0.003 |
| ALBERT | 84 | GPT-5.3 Chat | Random | 0.877 (0.031) | 0.881 (0.029) | 0.004 |
| ALBERT | 84 | Qwen3.6‑Plus | Random | 0.884 (0.019) | 0.887 (0.013) | 0.002 |
| BERT | 84 | Human | Stratified | 0.924 (0.025) | 0.923 (0.026) | 0.000 |
| BERT | 84 | Human | Random | 0.922 (0.037) | 0.926 (0.035) | 0.004 |
| BERT | 84 | Gemma 4 | Random | 0.898 (0.010) | 0.904 (0.010) | 0.006 |
| BERT | 84 | GPT-5.3 Chat | Random | 0.898 (0.007) | 0.903 (0.005) | 0.005 |
| BERT | 84 | Qwen3.6‑Plus | Random | 0.896 (0.019) | 0.900 (0.018) | 0.004 |
| Bio_ClinicalBERT | 84 | Human | Stratified | 0.944 (0.029) | 0.946 (0.029) | 0.002 |
| Bio_ClinicalBERT | 84 | Human | Random | 0.928 (0.014) | 0.932 (0.014) | 0.005 |
| Bio_ClinicalBERT | 84 | Gemma 4 | Random | 0.901 (0.012) | 0.908 (0.014) | 0.007 |
| Bio_ClinicalBERT | 84 | GPT-5.3 Chat | Random | 0.911 (0.013) | 0.915 (0.013) | 0.004 |
| Bio_ClinicalBERT | 84 | Qwen3.6‑Plus | Random | 0.896 (0.026) | 0.900 (0.025) | 0.004 |
| ALBERT | 126 | Human | Stratified | 0.963 (0.021) | 0.964 (0.021) | 0.002 |
| ALBERT | 126 | Human | Random | 0.945 (0.022) | 0.945 (0.021) | 0.000 |
| ALBERT | 126 | Gemma 4 | Random | 0.880 (0.032) | 0.884 (0.033) | 0.004 |
| ALBERT | 126 | GPT-5.3 Chat | Random | 0.902 (0.014) | 0.904 (0.017) | 0.002 |
| ALBERT | 126 | Qwen3.6‑Plus | Random | 0.884 (0.010) | 0.889 (0.010) | 0.005 |
| BERT | 126 | Human | Stratified | 0.947 (0.037) | 0.947 (0.033) | 0.001 |
| BERT | 126 | Human | Random | 0.914 (0.023) | 0.917 (0.023) | 0.003 |
| BERT | 126 | Gemma 4 | Random | 0.867 (0.035) | 0.871 (0.036) | 0.004 |
| BERT | 126 | GPT-5.3 Chat | Random | 0.884 (0.026) | 0.889 (0.024) | 0.005 |
| BERT | 126 | Qwen3.6‑Plus | Random | 0.846 (0.049) | 0.848 (0.053) | 0.002 |
| Bio_ClinicalBERT | 126 | Human | Stratified | 0.953 (0.033) | 0.954 (0.031) | 0.001 |
| Bio_ClinicalBERT | 126 | Human | Random | 0.921 (0.029) | 0.925 (0.027) | 0.004 |
| Bio_ClinicalBERT | 126 | Gemma 4 | Random | 0.903 (0.000) | 0.908 (0.002) | 0.005 |
| Bio_ClinicalBERT | 126 | GPT-5.3 Chat | Random | 0.907 (0.013) | 0.911 (0.013) | 0.004 |
| Bio_ClinicalBERT | 126 | Qwen3.6‑Plus | Random | 0.887 (0.019) | 0.893 (0.018) | 0.006 |
| ALBERT | 168 | Human | Stratified | 0.976 (0.004) | 0.977 (0.004) | 0.001 |
| ALBERT | 168 | Human | Random | 0.968 (0.008) | 0.969 (0.007) | 0.001 |
| ALBERT | 168 | Gemma 4 | Random | 0.868 (0.058) | 0.872 (0.056) | 0.004 |
| ALBERT | 168 | GPT-5.3 Chat | Random | 0.893 (0.032) | 0.897 (0.031) | 0.004 |
| ALBERT | 168 | Qwen3.6‑Plus | Random | 0.922 (0.021) | 0.921 (0.022) | -0.001 |
| BERT | 168 | Human | Stratified | 0.965 (0.006) | 0.965 (0.006) | 0.000 |
| BERT | 168 | Human | Random | 0.929 (0.035) | 0.933 (0.034) | 0.004 |
| BERT | 168 | Gemma 4 | Random | 0.888 (0.023) | 0.890 (0.025) | 0.003 |
| BERT | 168 | GPT-5.3 Chat | Random | 0.879 (0.015) | 0.883 (0.013) | 0.004 |
| BERT | 168 | Qwen3.6‑Plus | Random | 0.872 (0.017) | 0.875 (0.019) | 0.003 |
| Bio_ClinicalBERT | 168 | Human | Stratified | 0.967 (0.011) | 0.967 (0.013) | 0.000 |
| Bio_ClinicalBERT | 168 | Human | Random | 0.954 (0.006) | 0.955 (0.008) | 0.001 |
| Bio_ClinicalBERT | 168 | Gemma 4 | Random | 0.898 (0.010) | 0.904 (0.010) | 0.006 |
| Bio_ClinicalBERT | 168 | GPT-5.3 Chat | Random | 0.901 (0.008) | 0.906 (0.007) | 0.005 |
| Bio_ClinicalBERT | 168 | Qwen3.6‑Plus | Random | 0.892 (0.006) | 0.896 (0.006) | 0.005 |
| ALBERT | 210 | Human | Stratified | 0.972 (0.007) | 0.973 (0.007) | 0.002 |
| ALBERT | 210 | Human | Random | 0.969 (0.011) | 0.969 (0.014) | 0.000 |
| ALBERT | 210 | Gemma 4 | Random | 0.902 (0.016) | 0.907 (0.016) | 0.005 |
| ALBERT | 210 | GPT-5.3 Chat | Random | 0.872 (0.019) | 0.876 (0.016) | 0.004 |
| ALBERT | 210 | Qwen3.6‑Plus | Random | 0.880 (0.012) | 0.882 (0.010) | 0.002 |
| BERT | 210 | Human | Stratified | 0.976 (0.007) | 0.977 (0.005) | 0.000 |
| BERT | 210 | Human | Random | 0.957 (0.012) | 0.958 (0.012) | 0.000 |
| BERT | 210 | Gemma 4 | Random | 0.885 (0.019) | 0.888 (0.020) | 0.003 |
| BERT | 210 | GPT-5.3 Chat | Random | 0.863 (0.021) | 0.865 (0.021) | 0.002 |
| BERT | 210 | Qwen3.6‑Plus | Random | 0.866 (0.046) | 0.867 (0.044) | 0.001 |
| Bio_ClinicalBERT | 210 | Human | Stratified | 0.961 (0.003) | 0.962 (0.003) | 0.001 |
| Bio_ClinicalBERT | 210 | Human | Random | 0.949 (0.019) | 0.949 (0.018) | 0.001 |
| Bio_ClinicalBERT | 210 | Gemma 4 | Random | 0.897 (0.006) | 0.905 (0.005) | 0.007 |
| Bio_ClinicalBERT | 210 | GPT-5.3 Chat | Random | 0.896 (0.017) | 0.901 (0.015) | 0.004 |
| Bio_ClinicalBERT | 210 | Qwen3.6‑Plus | Random | 0.898 (0.017) | 0.904 (0.015) | 0.006 |
| ALBERT | 252 | Human | Stratified | 0.970 (0.015) | 0.971 (0.016) | 0.001 |
| ALBERT | 252 | Human | Random | 0.964 (0.008) | 0.963 (0.010) | -0.001 |
| ALBERT | 252 | Gemma 4 | Random | 0.912 (0.034) | 0.915 (0.034) | 0.003 |
| ALBERT | 252 | GPT-5.3 Chat | Random | 0.901 (0.052) | 0.903 (0.047) | 0.001 |
| ALBERT | 252 | Qwen3.6‑Plus | Random | 0.897 (0.077) | 0.896 (0.078) | -0.001 |
| BERT | 252 | Human | Stratified | 0.977 (0.003) | 0.976 (0.002) | -0.001 |
| BERT | 252 | Human | Random | 0.959 (0.014) | 0.958 (0.013) | 0.000 |
| BERT | 252 | Gemma 4 | Random | 0.860 (0.028) | 0.864 (0.028) | 0.004 |
| BERT | 252 | GPT-5.3 Chat | Random | 0.882 (0.005) | 0.889 (0.004) | 0.006 |
| BERT | 252 | Qwen3.6‑Plus | Random | 0.919 (0.015) | 0.921 (0.015) | 0.002 |
| Bio_ClinicalBERT | 252 | Human | Stratified | 0.962 (0.008) | 0.963 (0.007) | 0.001 |
| Bio_ClinicalBERT | 252 | Human | Random | 0.950 (0.003) | 0.950 (0.005) | 0.000 |
| Bio_ClinicalBERT | 252 | Gemma 4 | Random | 0.901 (0.041) | 0.905 (0.040) | 0.004 |
| Bio_ClinicalBERT | 252 | GPT-5.3 Chat | Random | 0.905 (0.002) | 0.909 (0.002) | 0.004 |
| Bio_ClinicalBERT | 252 | Qwen3.6‑Plus | Random | 0.926 (0.020) | 0.931 (0.020) | 0.005 |
| ALBERT | 294 | Human | Stratified | 0.977 (0.002) | 0.980 (0.002) | 0.003 |
| ALBERT | 294 | Human | Random | 0.963 (0.014) | 0.963 (0.014) | 0.000 |
| ALBERT | 294 | Gemma 4 | Random | 0.913 (0.043) | 0.916 (0.041) | 0.003 |
| ALBERT | 294 | GPT-5.3 Chat | Random | 0.924 (0.027) | 0.923 (0.026) | -0.001 |
| ALBERT | 294 | Qwen3.6‑Plus | Random | 0.919 (0.010) | 0.920 (0.010) | 0.001 |
| BERT | 294 | Human | Stratified | 0.975 (0.011) | 0.973 (0.011) | -0.001 |
| BERT | 294 | Human | Random | 0.967 (0.005) | 0.969 (0.003) | 0.002 |
| BERT | 294 | Gemma 4 | Random | 0.869 (0.011) | 0.873 (0.011) | 0.004 |
| BERT | 294 | GPT-5.3 Chat | Random | 0.871 (0.021) | 0.873 (0.022) | 0.003 |
| BERT | 294 | Qwen3.6‑Plus | Random | 0.911 (0.048) | 0.913 (0.048) | 0.002 |
| Bio_ClinicalBERT | 294 | Human | Stratified | 0.965 (0.012) | 0.966 (0.014) | 0.001 |
| Bio_ClinicalBERT | 294 | Human | Random | 0.942 (0.018) | 0.944 (0.016) | 0.003 |
| Bio_ClinicalBERT | 294 | Gemma 4 | Random | 0.906 (0.022) | 0.912 (0.022) | 0.006 |
| Bio_ClinicalBERT | 294 | GPT-5.3 Chat | Random | 0.919 (0.023) | 0.922 (0.022) | 0.003 |
| Bio_ClinicalBERT | 294 | Qwen3.6‑Plus | Random | 0.913 (0.028) | 0.918 (0.025) | 0.004 |
| ALBERT | 336 | Human | Stratified | 0.984 (0.005) | 0.986 (0.005) | 0.002 |
| ALBERT | 336 | Human | Random | 0.974 (0.002) | 0.976 (0.001) | 0.002 |
| ALBERT | 336 | Gemma 4 | Random | 0.915 (0.015) | 0.921 (0.014) | 0.005 |
| ALBERT | 336 | GPT-5.3 Chat | Random | 0.897 (0.014) | 0.899 (0.015) | 0.002 |
| ALBERT | 336 | Qwen3.6‑Plus | Random | 0.901 (0.010) | 0.904 (0.010) | 0.003 |
| BERT | 336 | Human | Stratified | 0.973 (0.002) | 0.974 (0.002) | 0.001 |
| BERT | 336 | Human | Random | 0.958 (0.009) | 0.962 (0.005) | 0.003 |
| BERT | 336 | Gemma 4 | Random | 0.867 (0.050) | 0.870 (0.050) | 0.003 |
| BERT | 336 | GPT-5.3 Chat | Random | 0.874 (0.032) | 0.875 (0.029) | 0.001 |
| BERT | 336 | Qwen3.6‑Plus | Random | 0.894 (0.011) | 0.898 (0.012) | 0.003 |
| Bio_ClinicalBERT | 336 | Human | Stratified | 0.973 (0.004) | 0.974 (0.003) | 0.000 |
| Bio_ClinicalBERT | 336 | Human | Random | 0.957 (0.010) | 0.958 (0.010) | 0.001 |
| Bio_ClinicalBERT | 336 | Gemma 4 | Random | 0.913 (0.016) | 0.917 (0.014) | 0.004 |
| Bio_ClinicalBERT | 336 | GPT-5.3 Chat | Random | 0.921 (0.005) | 0.925 (0.006) | 0.003 |
| Bio_ClinicalBERT | 336 | Qwen3.6‑Plus | Random | 0.919 (0.027) | 0.920 (0.024) | 0.001 |
| ALBERT | 378 | Human | Stratified | 0.980 (0.004) | 0.980 (0.004) | 0.000 |
| ALBERT | 378 | Human | Random | 0.969 (0.007) | 0.969 (0.009) | 0.000 |
| ALBERT | 378 | Gemma 4 | Random | 0.901 (0.008) | 0.909 (0.009) | 0.007 |
| ALBERT | 378 | GPT-5.3 Chat | Random | 0.942 (0.009) | 0.943 (0.007) | 0.000 |
| ALBERT | 378 | Qwen3.6‑Plus | Random | 0.902 (0.002) | 0.905 (0.001) | 0.004 |
| BERT | 378 | Human | Stratified | 0.978 (0.006) | 0.979 (0.005) | 0.001 |
| BERT | 378 | Human | Random | 0.948 (0.023) | 0.949 (0.020) | 0.002 |
| BERT | 378 | Gemma 4 | Random | 0.906 (0.014) | 0.913 (0.013) | 0.006 |
| BERT | 378 | GPT-5.3 Chat | Random | 0.922 (0.021) | 0.924 (0.016) | 0.002 |
| BERT | 378 | Qwen3.6‑Plus | Random | 0.909 (0.018) | 0.913 (0.017) | 0.003 |
| Bio_ClinicalBERT | 378 | Human | Stratified | 0.972 (0.004) | 0.973 (0.004) | 0.001 |
| Bio_ClinicalBERT | 378 | Human | Random | 0.952 (0.024) | 0.954 (0.020) | 0.001 |
| Bio_ClinicalBERT | 378 | Gemma 4 | Random | 0.922 (0.012) | 0.925 (0.012) | 0.002 |
| Bio_ClinicalBERT | 378 | GPT-5.3 Chat | Random | 0.919 (0.026) | 0.920 (0.025) | 0.000 |
| Bio_ClinicalBERT | 378 | Qwen3.6‑Plus | Random | 0.913 (0.025) | 0.915 (0.023) | 0.002 |
| ALBERT | 420 | Human | Stratified | 0.972 (0.014) | 0.971 (0.015) | 0.000 |
| ALBERT | 420 | Human | Random | 0.977 (0.010) | 0.977 (0.010) | 0.000 |
| ALBERT | 420 | Gemma 4 | Random | 0.939 (0.043) | 0.941 (0.039) | 0.002 |
| ALBERT | 420 | GPT-5.3 Chat | Random | 0.907 (0.030) | 0.906 (0.027) | -0.001 |
| ALBERT | 420 | Qwen3.6‑Plus | Random | 0.923 (0.031) | 0.924 (0.028) | 0.001 |
| BERT | 420 | Human | Stratified | 0.981 (0.004) | 0.982 (0.002) | 0.001 |
| BERT | 420 | Human | Random | 0.962 (0.014) | 0.960 (0.015) | -0.001 |
| BERT | 420 | Gemma 4 | Random | 0.896 (0.014) | 0.903 (0.016) | 0.007 |
| BERT | 420 | GPT-5.3 Chat | Random | 0.921 (0.032) | 0.923 (0.029) | 0.002 |
| BERT | 420 | Qwen3.6‑Plus | Random | 0.903 (0.049) | 0.907 (0.047) | 0.004 |
| Bio_ClinicalBERT | 420 | Human | Stratified | 0.973 (0.004) | 0.974 (0.004) | 0.001 |
| Bio_ClinicalBERT | 420 | Human | Random | 0.955 (0.014) | 0.958 (0.014) | 0.003 |
| Bio_ClinicalBERT | 420 | Gemma 4 | Random | 0.924 (0.017) | 0.930 (0.016) | 0.006 |
| Bio_ClinicalBERT | 420 | GPT-5.3 Chat | Random | 0.926 (0.014) | 0.926 (0.015) | 0.000 |
| Bio_ClinicalBERT | 420 | Qwen3.6‑Plus | Random | 0.915 (0.029) | 0.917 (0.028) | 0.002 |
| ALBERT | 462 | Human | Stratified | 0.983 (0.002) | 0.984 (0.002) | 0.001 |
| ALBERT | 462 | Human | Random | 0.964 (0.021) | 0.965 (0.020) | 0.001 |
| ALBERT | 462 | Gemma 4 | Random | 0.899 (0.012) | 0.906 (0.013) | 0.007 |
| ALBERT | 462 | GPT-5.3 Chat | Random | 0.933 (0.036) | 0.930 (0.037) | -0.003 |
| ALBERT | 462 | Qwen3.6‑Plus | Random | 0.935 (0.011) | 0.933 (0.011) | -0.001 |
| BERT | 462 | Human | Stratified | 0.978 (0.005) | 0.977 (0.005) | -0.002 |
| BERT | 462 | Human | Random | 0.949 (0.018) | 0.950 (0.016) | 0.001 |
| BERT | 462 | Gemma 4 | Random | 0.889 (0.025) | 0.892 (0.025) | 0.004 |
| BERT | 462 | GPT-5.3 Chat | Random | 0.914 (0.027) | 0.916 (0.029) | 0.001 |
| BERT | 462 | Qwen3.6‑Plus | Random | 0.922 (0.025) | 0.924 (0.022) | 0.002 |
| Bio_ClinicalBERT | 462 | Human | Stratified | 0.973 (0.008) | 0.974 (0.007) | 0.001 |
| Bio_ClinicalBERT | 462 | Human | Random | 0.957 (0.006) | 0.956 (0.007) | -0.001 |
| Bio_ClinicalBERT | 462 | Gemma 4 | Random | 0.914 (0.032) | 0.917 (0.030) | 0.003 |
| Bio_ClinicalBERT | 462 | GPT-5.3 Chat | Random | 0.934 (0.031) | 0.934 (0.032) | 0.000 |
| Bio_ClinicalBERT | 462 | Qwen3.6‑Plus | Random | 0.932 (0.015) | 0.934 (0.011) | 0.002 |
| ALBERT | 504 | Human | Stratified | 0.981 (0.002) | 0.981 (0.003) | 0.001 |
| ALBERT | 504 | Human | Random | 0.974 (0.011) | 0.974 (0.011) | 0.001 |
| ALBERT | 504 | Gemma 4 | Random | 0.957 (0.011) | 0.960 (0.007) | 0.003 |
| ALBERT | 504 | GPT-5.3 Chat | Random | 0.912 (0.023) | 0.911 (0.019) | 0.000 |
| ALBERT | 504 | Qwen3.6‑Plus | Random | 0.923 (0.044) | 0.922 (0.040) | -0.001 |
| BERT | 504 | Human | Stratified | 0.982 (0.002) | 0.981 (0.002) | -0.001 |
| BERT | 504 | Human | Random | 0.954 (0.021) | 0.955 (0.018) | 0.001 |
| BERT | 504 | Gemma 4 | Random | 0.940 (0.003) | 0.945 (0.004) | 0.005 |
| BERT | 504 | GPT-5.3 Chat | Random | 0.884 (0.018) | 0.886 (0.015) | 0.002 |
| BERT | 504 | Qwen3.6‑Plus | Random | 0.911 (0.046) | 0.912 (0.042) | 0.001 |
| Bio_ClinicalBERT | 504 | Human | Stratified | 0.971 (0.003) | 0.972 (0.002) | 0.001 |
| Bio_ClinicalBERT | 504 | Human | Random | 0.971 (0.003) | 0.970 (0.004) | -0.001 |
| Bio_ClinicalBERT | 504 | Gemma 4 | Random | 0.916 (0.029) | 0.920 (0.025) | 0.004 |
| Bio_ClinicalBERT | 504 | GPT-5.3 Chat | Random | 0.900 (0.003) | 0.902 (0.003) | 0.002 |
| Bio_ClinicalBERT | 504 | Qwen3.6‑Plus | Random | 0.935 (0.017) | 0.935 (0.014) | 0.000 |
| ALBERT | 546 | Human | Stratified | 0.977 (0.009) | 0.979 (0.009) | 0.002 |
| ALBERT | 546 | Human | Random | 0.969 (0.004) | 0.969 (0.004) | 0.000 |
| ALBERT | 546 | Gemma 4 | Random | 0.931 (0.042) | 0.932 (0.046) | 0.002 |
| ALBERT | 546 | GPT-5.3 Chat | Random | 0.958 (0.023) | 0.960 (0.019) | 0.002 |
| ALBERT | 546 | Qwen3.6‑Plus | Random | 0.951 (0.005) | 0.950 (0.009) | -0.001 |
| BERT | 546 | Human | Stratified | 0.968 (0.002) | 0.969 (0.003) | 0.001 |
| BERT | 546 | Human | Random | 0.954 (0.031) | 0.957 (0.027) | 0.003 |
| BERT | 546 | Gemma 4 | Random | 0.917 (0.039) | 0.921 (0.035) | 0.004 |
| BERT | 546 | GPT-5.3 Chat | Random | 0.917 (0.027) | 0.919 (0.026) | 0.002 |
| BERT | 546 | Qwen3.6‑Plus | Random | 0.893 (0.030) | 0.896 (0.033) | 0.002 |
| Bio_ClinicalBERT | 546 | Human | Stratified | 0.975 (0.004) | 0.976 (0.005) | 0.001 |
| Bio_ClinicalBERT | 546 | Human | Random | 0.966 (0.009) | 0.966 (0.010) | 0.000 |
| Bio_ClinicalBERT | 546 | Gemma 4 | Random | 0.918 (0.024) | 0.924 (0.025) | 0.006 |
| Bio_ClinicalBERT | 546 | GPT-5.3 Chat | Random | 0.933 (0.027) | 0.934 (0.026) | 0.001 |
| Bio_ClinicalBERT | 546 | Qwen3.6‑Plus | Random | 0.921 (0.033) | 0.924 (0.029) | 0.003 |
| ALBERT | 588 | Human | Stratified | 0.980 (0.005) | 0.980 (0.004) | 0.000 |
| ALBERT | 588 | Human | Random | 0.974 (0.013) | 0.974 (0.014) | -0.001 |
| ALBERT | 588 | Gemma 4 | Random | 0.936 (0.008) | 0.939 (0.004) | 0.003 |
| ALBERT | 588 | GPT-5.3 Chat | Random | 0.948 (0.023) | 0.948 (0.019) | 0.000 |
| ALBERT | 588 | Qwen3.6‑Plus | Random | 0.937 (0.008) | 0.936 (0.007) | -0.002 |
| BERT | 588 | Human | Stratified | 0.980 (0.004) | 0.982 (0.004) | 0.001 |
| BERT | 588 | Human | Random | 0.954 (0.023) | 0.957 (0.021) | 0.003 |
| BERT | 588 | Gemma 4 | Random | 0.927 (0.016) | 0.933 (0.013) | 0.006 |
| BERT | 588 | GPT-5.3 Chat | Random | 0.937 (0.024) | 0.938 (0.019) | 0.000 |
| BERT | 588 | Qwen3.6‑Plus | Random | 0.902 (0.023) | 0.904 (0.022) | 0.002 |
| Bio_ClinicalBERT | 588 | Human | Stratified | 0.977 (0.005) | 0.978 (0.005) | 0.001 |
| Bio_ClinicalBERT | 588 | Human | Random | 0.969 (0.006) | 0.970 (0.004) | 0.001 |
| Bio_ClinicalBERT | 588 | Gemma 4 | Random | 0.937 (0.017) | 0.941 (0.014) | 0.004 |
| Bio_ClinicalBERT | 588 | GPT-5.3 Chat | Random | 0.939 (0.033) | 0.939 (0.030) | 0.000 |
| Bio_ClinicalBERT | 588 | Qwen3.6‑Plus | Random | 0.946 (0.018) | 0.947 (0.014) | 0.002 |
| ALBERT | 630 | Human | Stratified | 0.979 (0.008) | 0.979 (0.009) | 0.000 |
| ALBERT | 630 | Human | Random | 0.963 (0.016) | 0.963 (0.015) | 0.000 |
| ALBERT | 630 | Gemma 4 | Random | 0.947 (0.042) | 0.949 (0.038) | 0.002 |
| ALBERT | 630 | GPT-5.3 Chat | Random | 0.951 (0.015) | 0.947 (0.016) | -0.003 |
| ALBERT | 630 | Qwen3.6‑Plus | Random | 0.947 (0.019) | 0.945 (0.020) | -0.003 |
| BERT | 630 | Human | Stratified | 0.979 (0.007) | 0.979 (0.008) | 0.000 |
| BERT | 630 | Human | Random | 0.965 (0.011) | 0.966 (0.013) | 0.000 |
| BERT | 630 | Gemma 4 | Random | 0.920 (0.029) | 0.922 (0.024) | 0.002 |
| BERT | 630 | GPT-5.3 Chat | Random | 0.902 (0.029) | 0.905 (0.030) | 0.003 |
| BERT | 630 | Qwen3.6‑Plus | Random | 0.925 (0.025) | 0.931 (0.027) | 0.006 |
| Bio_ClinicalBERT | 630 | Human | Stratified | 0.971 (0.009) | 0.971 (0.009) | 0.000 |
| Bio_ClinicalBERT | 630 | Human | Random | 0.967 (0.007) | 0.967 (0.009) | 0.000 |
| Bio_ClinicalBERT | 630 | Gemma 4 | Random | 0.936 (0.029) | 0.941 (0.025) | 0.004 |
| Bio_ClinicalBERT | 630 | GPT-5.3 Chat | Random | 0.931 (0.033) | 0.933 (0.031) | 0.002 |
| Bio_ClinicalBERT | 630 | Qwen3.6‑Plus | Random | 0.944 (0.021) | 0.944 (0.019) | 0.000 |
| ALBERT | 672 | Human | Stratified | 0.986 (0.001) | 0.987 (0.001) | 0.001 |
| ALBERT | 672 | Human | Random | 0.976 (0.013) | 0.975 (0.013) | -0.001 |
| ALBERT | 672 | Gemma 4 | Random | 0.946 (0.016) | 0.946 (0.016) | -0.001 |
| ALBERT | 672 | GPT-5.3 Chat | Random | 0.931 (0.042) | 0.930 (0.038) | -0.001 |
| ALBERT | 672 | Qwen3.6‑Plus | Random | 0.941 (0.003) | 0.939 (0.005) | -0.002 |
| BERT | 672 | Human | Stratified | 0.964 (0.020) | 0.966 (0.020) | 0.002 |
| BERT | 672 | Human | Random | 0.969 (0.017) | 0.969 (0.017) | 0.000 |
| BERT | 672 | Gemma 4 | Random | 0.909 (0.041) | 0.913 (0.039) | 0.004 |
| BERT | 672 | GPT-5.3 Chat | Random | 0.898 (0.024) | 0.897 (0.020) | 0.000 |
| BERT | 672 | Qwen3.6‑Plus | Random | 0.911 (0.021) | 0.913 (0.020) | 0.002 |
| Bio_ClinicalBERT | 672 | Human | Stratified | 0.967 (0.013) | 0.968 (0.013) | 0.001 |
| Bio_ClinicalBERT | 672 | Human | Random | 0.973 (0.004) | 0.974 (0.004) | 0.001 |
| Bio_ClinicalBERT | 672 | Gemma 4 | Random | 0.940 (0.022) | 0.942 (0.020) | 0.002 |
| Bio_ClinicalBERT | 672 | GPT-5.3 Chat | Random | 0.932 (0.032) | 0.932 (0.029) | 0.000 |
| Bio_ClinicalBERT | 672 | Qwen3.6‑Plus | Random | 0.940 (0.012) | 0.938 (0.011) | -0.002 |
| ALBERT | 714 | Human | Stratified | 0.982 (0.003) | 0.982 (0.003) | 0.000 |
| ALBERT | 714 | Human | Random | 0.976 (0.004) | 0.977 (0.005) | 0.001 |
| ALBERT | 714 | Gemma 4 | Random | 0.931 (0.025) | 0.932 (0.026) | 0.001 |
| ALBERT | 714 | GPT-5.3 Chat | Random | 0.960 (0.016) | 0.958 (0.016) | -0.002 |
| ALBERT | 714 | Qwen3.6‑Plus | Random | 0.924 (0.036) | 0.922 (0.037) | -0.002 |
| BERT | 714 | Human | Stratified | 0.978 (0.008) | 0.978 (0.007) | 0.000 |
| BERT | 714 | Human | Random | 0.972 (0.003) | 0.971 (0.003) | -0.001 |
| BERT | 714 | Gemma 4 | Random | 0.924 (0.036) | 0.925 (0.034) | 0.001 |
| BERT | 714 | GPT-5.3 Chat | Random | 0.912 (0.026) | 0.915 (0.024) | 0.003 |
| BERT | 714 | Qwen3.6‑Plus | Random | 0.942 (0.024) | 0.943 (0.022) | 0.001 |
| Bio_ClinicalBERT | 714 | Human | Stratified | 0.972 (0.008) | 0.973 (0.007) | 0.001 |
| Bio_ClinicalBERT | 714 | Human | Random | 0.971 (0.008) | 0.971 (0.010) | 0.000 |
| Bio_ClinicalBERT | 714 | Gemma 4 | Random | 0.929 (0.008) | 0.931 (0.008) | 0.002 |
| Bio_ClinicalBERT | 714 | GPT-5.3 Chat | Random | 0.963 (0.008) | 0.962 (0.010) | -0.001 |
| Bio_ClinicalBERT | 714 | Qwen3.6‑Plus | Random | 0.953 (0.020) | 0.951 (0.019) | -0.001 |
| ALBERT | 756 | Human | Stratified | 0.979 (0.009) | 0.980 (0.009) | 0.001 |
| ALBERT | 756 | Human | Random | 0.976 (0.009) | 0.980 (0.008) | 0.003 |
| ALBERT | 756 | Gemma 4 | Random | 0.931 (0.010) | 0.934 (0.013) | 0.003 |
| ALBERT | 756 | GPT-5.3 Chat | Random | 0.907 (0.005) | 0.909 (0.005) | 0.002 |
| ALBERT | 756 | Qwen3.6‑Plus | Random | 0.954 (0.008) | 0.952 (0.008) | -0.002 |
| BERT | 756 | Human | Stratified | 0.985 (0.001) | 0.985 (0.001) | -0.001 |
| BERT | 756 | Human | Random | 0.977 (0.002) | 0.976 (0.001) | -0.001 |
| BERT | 756 | Gemma 4 | Random | 0.913 (0.003) | 0.919 (0.005) | 0.006 |
| BERT | 756 | GPT-5.3 Chat | Random | 0.934 (0.030) | 0.935 (0.027) | 0.001 |
| BERT | 756 | Qwen3.6‑Plus | Random | 0.958 (0.012) | 0.957 (0.014) | -0.002 |
| Bio_ClinicalBERT | 756 | Human | Stratified | 0.973 (0.005) | 0.973 (0.007) | 0.000 |
| Bio_ClinicalBERT | 756 | Human | Random | 0.971 (0.007) | 0.973 (0.005) | 0.001 |
| Bio_ClinicalBERT | 756 | Gemma 4 | Random | 0.918 (0.004) | 0.923 (0.007) | 0.005 |
| Bio_ClinicalBERT | 756 | GPT-5.3 Chat | Random | 0.950 (0.005) | 0.947 (0.004) | -0.003 |
| Bio_ClinicalBERT | 756 | Qwen3.6‑Plus | Random | 0.942 (0.014) | 0.940 (0.015) | -0.002 |
| ALBERT | 798 | Human | Stratified | 0.984 (0.006) | 0.984 (0.006) | 0.001 |
| ALBERT | 798 | Human | Random | 0.977 (0.004) | 0.977 (0.005) | 0.000 |
| ALBERT | 798 | Gemma 4 | Random | 0.949 (0.019) | 0.950 (0.018) | 0.001 |
| ALBERT | 798 | GPT-5.3 Chat | Random | 0.957 (0.025) | 0.956 (0.026) | -0.001 |
| ALBERT | 798 | Qwen3.6‑Plus | Random | 0.954 (0.004) | 0.953 (0.002) | -0.001 |
| BERT | 798 | Human | Stratified | 0.976 (0.014) | 0.976 (0.014) | 0.000 |
| BERT | 798 | Human | Random | 0.978 (0.007) | 0.978 (0.008) | 0.000 |
| BERT | 798 | Gemma 4 | Random | 0.926 (0.030) | 0.929 (0.027) | 0.003 |
| BERT | 798 | GPT-5.3 Chat | Random | 0.940 (0.036) | 0.941 (0.035) | 0.001 |
| BERT | 798 | Qwen3.6‑Plus | Random | 0.945 (0.020) | 0.946 (0.018) | 0.000 |
| Bio_ClinicalBERT | 798 | Human | Stratified | 0.976 (0.007) | 0.977 (0.008) | 0.001 |
| Bio_ClinicalBERT | 798 | Human | Random | 0.969 (0.008) | 0.970 (0.008) | 0.001 |
| Bio_ClinicalBERT | 798 | Gemma 4 | Random | 0.944 (0.018) | 0.947 (0.016) | 0.002 |
| Bio_ClinicalBERT | 798 | GPT-5.3 Chat | Random | 0.961 (0.015) | 0.960 (0.017) | -0.001 |
| Bio_ClinicalBERT | 798 | Qwen3.6‑Plus | Random | 0.953 (0.007) | 0.951 (0.006) | -0.002 |
| ALBERT | 840 | Human | Stratified | 0.982 (0.004) | 0.983 (0.004) | 0.000 |
| ALBERT | 840 | Human | Random | 0.977 (0.013) | 0.979 (0.013) | 0.002 |
| ALBERT | 840 | Gemma 4 | Random | 0.951 (0.004) | 0.951 (0.008) | 0.001 |
| ALBERT | 840 | GPT-5.3 Chat | Random | 0.963 (0.013) | 0.961 (0.013) | -0.002 |
| ALBERT | 840 | Qwen3.6‑Plus | Random | 0.957 (0.012) | 0.957 (0.011) | 0.000 |
| BERT | 840 | Human | Stratified | 0.978 (0.009) | 0.977 (0.009) | -0.001 |
| BERT | 840 | Human | Random | 0.982 (0.005) | 0.982 (0.006) | 0.000 |
| BERT | 840 | Gemma 4 | Random | 0.911 (0.039) | 0.915 (0.040) | 0.004 |
| BERT | 840 | GPT-5.3 Chat | Random | 0.947 (0.022) | 0.949 (0.020) | 0.001 |
| BERT | 840 | Qwen3.6‑Plus | Random | 0.953 (0.016) | 0.952 (0.015) | -0.001 |
| Bio_ClinicalBERT | 840 | Human | Stratified | 0.982 (0.001) | 0.981 (0.001) | -0.001 |
| Bio_ClinicalBERT | 840 | Human | Random | 0.975 (0.000) | 0.976 (0.001) | 0.001 |
| Bio_ClinicalBERT | 840 | Gemma 4 | Random | 0.937 (0.025) | 0.939 (0.021) | 0.002 |
| Bio_ClinicalBERT | 840 | GPT-5.3 Chat | Random | 0.955 (0.021) | 0.954 (0.021) | -0.001 |
| Bio_ClinicalBERT | 840 | Qwen3.6‑Plus | Random | 0.964 (0.016) | 0.963 (0.016) | -0.001 |
| ALBERT | 1260 | Human | Stratified | 0.971 (0.004) | 0.969 (0.004) | -0.002 |
| ALBERT | 1260 | Human | Random | 0.983 (0.001) | 0.984 (0.002) | 0.001 |
| ALBERT | 1260 | Gemma 4 | Random | 0.960 (0.009) | 0.960 (0.010) | 0.001 |
| ALBERT | 1260 | GPT-5.3 Chat | Random | 0.969 (0.007) | 0.969 (0.007) | 0.000 |
| ALBERT | 1260 | Qwen3.6‑Plus | Random | 0.961 (0.004) | 0.960 (0.006) | -0.001 |
| BERT | 1260 | Human | Stratified | 0.977 (0.008) | 0.978 (0.007) | 0.000 |
| BERT | 1260 | Human | Random | 0.979 (0.008) | 0.980 (0.008) | 0.001 |
| BERT | 1260 | Gemma 4 | Random | 0.961 (0.011) | 0.961 (0.009) | 0.000 |
| BERT | 1260 | GPT-5.3 Chat | Random | 0.930 (0.064) | 0.931 (0.060) | 0.001 |
| BERT | 1260 | Qwen3.6‑Plus | Random | 0.938 (0.023) | 0.939 (0.021) | 0.002 |
| Bio_ClinicalBERT | 1260 | Human | Stratified | 0.976 (0.002) | 0.976 (0.002) | 0.000 |
| Bio_ClinicalBERT | 1260 | Human | Random | 0.977 (0.006) | 0.977 (0.006) | 0.000 |
| Bio_ClinicalBERT | 1260 | Gemma 4 | Random | 0.952 (0.024) | 0.953 (0.019) | 0.001 |
| Bio_ClinicalBERT | 1260 | GPT-5.3 Chat | Random | 0.972 (0.005) | 0.971 (0.005) | -0.001 |
| Bio_ClinicalBERT | 1260 | Qwen3.6‑Plus | Random | 0.961 (0.010) | 0.961 (0.009) | 0.001 |
| ALBERT | 1680 | Human | Stratified | 0.981 (0.007) | 0.981 (0.007) | 0.000 |
| ALBERT | 1680 | Human | Random | 0.985 (0.002) | 0.987 (0.003) | 0.002 |
| ALBERT | 1680 | Gemma 4 | Random | 0.956 (0.002) | 0.958 (0.006) | 0.002 |
| ALBERT | 1680 | GPT-5.3 Chat | Random | 0.976 (0.004) | 0.976 (0.004) | 0.000 |
| ALBERT | 1680 | Qwen3.6‑Plus | Random | 0.974 (0.006) | 0.974 (0.006) | 0.000 |
| BERT | 1680 | Human | Stratified | 0.981 (0.004) | 0.980 (0.004) | -0.001 |
| BERT | 1680 | Human | Random | 0.977 (0.010) | 0.976 (0.011) | -0.001 |
| BERT | 1680 | Gemma 4 | Random | 0.967 (0.016) | 0.966 (0.016) | -0.002 |
| BERT | 1680 | GPT-5.3 Chat | Random | 0.970 (0.005) | 0.968 (0.004) | -0.002 |
| BERT | 1680 | Qwen3.6‑Plus | Random | 0.962 (0.016) | 0.960 (0.015) | -0.002 |
| Bio_ClinicalBERT | 1680 | Human | Stratified | 0.981 (0.002) | 0.982 (0.002) | 0.000 |
| Bio_ClinicalBERT | 1680 | Human | Random | 0.981 (0.007) | 0.981 (0.006) | 0.001 |
| Bio_ClinicalBERT | 1680 | Gemma 4 | Random | 0.969 (0.003) | 0.970 (0.003) | 0.002 |
| Bio_ClinicalBERT | 1680 | GPT-5.3 Chat | Random | 0.975 (0.002) | 0.976 (0.005) | 0.001 |
| Bio_ClinicalBERT | 1680 | Qwen3.6‑Plus | Random | 0.965 (0.017) | 0.964 (0.016) | -0.001 |
| ALBERT | 2100 | Human | Stratified | 0.982 (0.002) | 0.984 (0.003) | 0.002 |
| ALBERT | 2100 | Human | Random | 0.987 (0.001) | 0.988 (0.001) | 0.001 |
| ALBERT | 2100 | Gemma 4 | Random | 0.970 (0.008) | 0.970 (0.008) | 0.000 |
| ALBERT | 2100 | GPT-5.3 Chat | Random | 0.972 (0.002) | 0.971 (0.003) | -0.001 |
| ALBERT | 2100 | Qwen3.6‑Plus | Random | 0.941 (0.004) | 0.940 (0.004) | -0.002 |
| BERT | 2100 | Human | Stratified | 0.981 (0.005) | 0.980 (0.005) | -0.001 |
| BERT | 2100 | Human | Random | 0.980 (0.005) | 0.980 (0.006) | 0.000 |
| BERT | 2100 | Gemma 4 | Random | 0.962 (0.008) | 0.961 (0.009) | -0.001 |
| BERT | 2100 | GPT-5.3 Chat | Random | 0.970 (0.004) | 0.969 (0.003) | 0.000 |
| BERT | 2100 | Qwen3.6‑Plus | Random | 0.930 (0.037) | 0.934 (0.035) | 0.003 |
| Bio_ClinicalBERT | 2100 | Human | Stratified | 0.983 (0.002) | 0.983 (0.001) | 0.001 |
| Bio_ClinicalBERT | 2100 | Human | Random | 0.983 (0.002) | 0.984 (0.002) | 0.000 |
| Bio_ClinicalBERT | 2100 | Gemma 4 | Random | 0.964 (0.002) | 0.963 (0.003) | -0.001 |
| Bio_ClinicalBERT | 2100 | GPT-5.3 Chat | Random | 0.968 (0.017) | 0.968 (0.016) | 0.000 |
| Bio_ClinicalBERT | 2100 | Qwen3.6‑Plus | Random | 0.964 (0.013) | 0.963 (0.013) | -0.001 |
| ALBERT | 2520 | Human | Stratified | 0.981 (0.006) | 0.980 (0.006) | -0.001 |
| ALBERT | 2520 | Human | Random | 0.987 (0.001) | 0.987 (0.001) | 0.001 |
| ALBERT | 2520 | Gemma 4 | Random | 0.965 (0.005) | 0.965 (0.006) | 0.000 |
| ALBERT | 2520 | GPT-5.3 Chat | Random | 0.966 (0.020) | 0.967 (0.017) | 0.002 |
| ALBERT | 2520 | Qwen3.6‑Plus | Random | 0.958 (0.013) | 0.958 (0.013) | 0.000 |
| BERT | 2520 | Human | Stratified | 0.977 (0.005) | 0.977 (0.005) | 0.000 |
| BERT | 2520 | Human | Random | 0.987 (0.002) | 0.987 (0.001) | 0.000 |
| BERT | 2520 | Gemma 4 | Random | 0.951 (0.026) | 0.954 (0.023) | 0.004 |
| BERT | 2520 | GPT-5.3 Chat | Random | 0.972 (0.005) | 0.970 (0.006) | -0.001 |
| BERT | 2520 | Qwen3.6‑Plus | Random | 0.935 (0.040) | 0.935 (0.036) | 0.001 |
| Bio_ClinicalBERT | 2520 | Human | Stratified | 0.983 (0.003) | 0.984 (0.005) | 0.000 |
| Bio_ClinicalBERT | 2520 | Human | Random | 0.986 (0.002) | 0.987 (0.002) | 0.001 |
| Bio_ClinicalBERT | 2520 | Gemma 4 | Random | 0.968 (0.006) | 0.969 (0.006) | 0.001 |
| Bio_ClinicalBERT | 2520 | GPT-5.3 Chat | Random | 0.966 (0.009) | 0.966 (0.010) | 0.000 |
| Bio_ClinicalBERT | 2520 | Qwen3.6‑Plus | Random | 0.959 (0.013) | 0.956 (0.013) | -0.003 |
| ALBERT | 2940 | Human | Stratified | 0.987 (0.004) | 0.987 (0.003) | 0.001 |
| ALBERT | 2940 | Human | Random | 0.986 (0.004) | 0.986 (0.004) | 0.000 |
| ALBERT | 2940 | Gemma 4 | Random | 0.968 (0.007) | 0.967 (0.009) | -0.001 |
| ALBERT | 2940 | GPT-5.3 Chat | Random | 0.981 (0.005) | 0.980 (0.005) | -0.001 |
| ALBERT | 2940 | Qwen3.6‑Plus | Random | 0.955 (0.016) | 0.955 (0.018) | 0.000 |
| BERT | 2940 | Human | Stratified | 0.989 (0.002) | 0.989 (0.001) | 0.001 |
| BERT | 2940 | Human | Random | 0.986 (0.002) | 0.987 (0.002) | 0.000 |
| BERT | 2940 | Gemma 4 | Random | 0.970 (0.014) | 0.968 (0.013) | -0.002 |
| BERT | 2940 | GPT-5.3 Chat | Random | 0.963 (0.003) | 0.962 (0.005) | -0.001 |
| BERT | 2940 | Qwen3.6‑Plus | Random | 0.955 (0.033) | 0.956 (0.030) | 0.001 |
| Bio_ClinicalBERT | 2940 | Human | Stratified | 0.985 (0.001) | 0.985 (0.001) | 0.000 |
| Bio_ClinicalBERT | 2940 | Human | Random | 0.985 (0.001) | 0.986 (0.001) | 0.001 |
| Bio_ClinicalBERT | 2940 | Gemma 4 | Random | 0.973 (0.003) | 0.973 (0.004) | 0.000 |
| Bio_ClinicalBERT | 2940 | GPT-5.3 Chat | Random | 0.971 (0.007) | 0.970 (0.007) | 0.000 |
| Bio_ClinicalBERT | 2940 | Qwen3.6‑Plus | Random | 0.966 (0.011) | 0.966 (0.012) | 0.000 |
| ALBERT | 3360 | Human | Stratified | 0.988 (0.001) | 0.988 (0.001) | 0.000 |
| ALBERT | 3360 | Human | Random | 0.984 (0.003) | 0.985 (0.003) | 0.001 |
| ALBERT | 3360 | Gemma 4 | Random | 0.961 (0.007) | 0.959 (0.006) | -0.002 |
| ALBERT | 3360 | GPT-5.3 Chat | Random | 0.956 (0.011) | 0.956 (0.013) | -0.001 |
| ALBERT | 3360 | Qwen3.6‑Plus | Random | 0.963 (0.005) | 0.963 (0.005) | 0.000 |
| BERT | 3360 | Human | Stratified | 0.987 (0.003) | 0.987 (0.003) | 0.000 |
| BERT | 3360 | Human | Random | 0.988 (0.001) | 0.988 (0.001) | 0.000 |
| BERT | 3360 | Gemma 4 | Random | 0.941 (0.030) | 0.945 (0.028) | 0.004 |
| BERT | 3360 | GPT-5.3 Chat | Random | 0.963 (0.023) | 0.963 (0.018) | 0.001 |
| BERT | 3360 | Qwen3.6‑Plus | Random | 0.960 (0.019) | 0.961 (0.017) | 0.000 |
| Bio_ClinicalBERT | 3360 | Human | Stratified | 0.984 (0.002) | 0.984 (0.001) | 0.000 |
| Bio_ClinicalBERT | 3360 | Human | Random | 0.984 (0.002) | 0.985 (0.002) | 0.001 |
| Bio_ClinicalBERT | 3360 | Gemma 4 | Random | 0.949 (0.019) | 0.951 (0.017) | 0.002 |
| Bio_ClinicalBERT | 3360 | GPT-5.3 Chat | Random | 0.970 (0.006) | 0.969 (0.006) | -0.001 |
| Bio_ClinicalBERT | 3360 | Qwen3.6‑Plus | Random | 0.967 (0.010) | 0.966 (0.012) | -0.001 |
| ALBERT | 3780 | Human | Stratified | 0.985 (0.005) | 0.985 (0.005) | 0.001 |
| ALBERT | 3780 | Human | Random | 0.984 (0.004) | 0.985 (0.005) | 0.001 |
| ALBERT | 3780 | Gemma 4 | Random | 0.958 (0.021) | 0.961 (0.020) | 0.003 |
| ALBERT | 3780 | GPT-5.3 Chat | Random | 0.973 (0.008) | 0.971 (0.009) | -0.002 |
| ALBERT | 3780 | Qwen3.6‑Plus | Random | 0.964 (0.012) | 0.964 (0.013) | 0.000 |
| BERT | 3780 | Human | Stratified | 0.976 (0.013) | 0.977 (0.012) | 0.000 |
| BERT | 3780 | Human | Random | 0.986 (0.003) | 0.986 (0.002) | -0.001 |
| BERT | 3780 | Gemma 4 | Random | 0.963 (0.026) | 0.962 (0.023) | -0.001 |
| BERT | 3780 | GPT-5.3 Chat | Random | 0.967 (0.005) | 0.966 (0.004) | -0.002 |
| BERT | 3780 | Qwen3.6‑Plus | Random | 0.946 (0.024) | 0.948 (0.020) | 0.002 |
| Bio_ClinicalBERT | 3780 | Human | Stratified | 0.985 (0.003) | 0.986 (0.002) | 0.001 |
| Bio_ClinicalBERT | 3780 | Human | Random | 0.985 (0.004) | 0.985 (0.004) | 0.000 |
| Bio_ClinicalBERT | 3780 | Gemma 4 | Random | 0.976 (0.004) | 0.976 (0.005) | 0.000 |
| Bio_ClinicalBERT | 3780 | GPT-5.3 Chat | Random | 0.975 (0.005) | 0.974 (0.006) | -0.001 |
| Bio_ClinicalBERT | 3780 | Qwen3.6‑Plus | Random | 0.952 (0.018) | 0.950 (0.016) | -0.002 |
| ALBERT | 4200 | Human | Stratified | 0.982 (0.011) | 0.982 (0.011) | 0.000 |
| ALBERT | 4200 | Human | Random | 0.977 (0.008) | 0.978 (0.008) | 0.000 |
| ALBERT | 4200 | Gemma 4 | Random | 0.970 (0.005) | 0.970 (0.005) | 0.000 |
| ALBERT | 4200 | GPT-5.3 Chat | Random | 0.972 (0.002) | 0.973 (0.002) | 0.001 |
| ALBERT | 4200 | Qwen3.6‑Plus | Random | 0.947 (0.011) | 0.946 (0.010) | -0.001 |
| BERT | 4200 | Human | Stratified | 0.981 (0.006) | 0.981 (0.007) | 0.000 |
| BERT | 4200 | Human | Random | 0.985 (0.008) | 0.985 (0.008) | 0.000 |
| BERT | 4200 | Gemma 4 | Random | 0.962 (0.017) | 0.960 (0.016) | -0.002 |
| BERT | 4200 | GPT-5.3 Chat | Random | 0.949 (0.018) | 0.951 (0.015) | 0.002 |
| BERT | 4200 | Qwen3.6‑Plus | Random | 0.924 (0.041) | 0.928 (0.037) | 0.004 |
| Bio_ClinicalBERT | 4200 | Human | Stratified | 0.984 (0.001) | 0.985 (0.002) | 0.001 |
| Bio_ClinicalBERT | 4200 | Human | Random | 0.984 (0.001) | 0.985 (0.002) | 0.001 |
| Bio_ClinicalBERT | 4200 | Gemma 4 | Random | 0.963 (0.018) | 0.963 (0.016) | -0.001 |
| Bio_ClinicalBERT | 4200 | GPT-5.3 Chat | Random | 0.969 (0.010) | 0.968 (0.009) | 0.000 |
| Bio_ClinicalBERT | 4200 | Qwen3.6‑Plus | Random | 0.967 (0.008) | 0.965 (0.009) | -0.002 |

**Table S2.**	Mean and sample standard deviation of keyword-level F1 scores across

three data-seed-specific runs for direct 5-shot and 10-shot large

language model inference and Transformer configurations. We

calculated F1 scores on the original test dataset and after excluding

test-set patients who also appeared in the training or validation set. This

descriptive analysis used no bootstrap resampling or hypothesis testing.

ΔF1: overlap-excluded mean F1 minus the original-test-dataset mean

F1; N: the number of few-shot examples or the training-data size.
