# Supplementary material for: Reducing Annotation Costs Using Knowledge Distillation for Label‑Efficient Negation Detection in Radiology Reports: Multisite Internal Validation Study
Source: JMIR Med Inform. 2026 Oct 22;14:e103912. doi: 10.2196/103912 (PMC13740815; doi:10.2196/103912)
Supplement: Supplementary file 3 [file medinform_v14i1e103912_app3.docx]

|  | A. Human-Distill |  |  |  |
| --- | --- | --- | --- | --- |
| N | Human-Distill | Gemma  (Human-Distilled) | GPT  (Human-Distilled) | Qwen  (Human-Distilled) |
| 42 | 0.011 (0.007 ~ 0.015) | 0.001 (-0.001 ~ 0.004) | 0.010 (0.005 ~ 0.014) | 0.022 (0.016 ~ 0.029) |
| 84 | 0.030 (0.022 ~ 0.037) | 0.030 (0.023 ~ 0.036) | 0.028 (0.020 ~ 0.036) | 0.031 (0.023 ~ 0.040) |
| 126 | 0.042 (0.031 ~ 0.053) | 0.043 (0.031 ~ 0.055) | 0.029 (0.020 ~ 0.038) | 0.054 (0.041 ~ 0.068) |
| 168 | 0.060 (0.045 ~ 0.075) | 0.065 (0.049 ~ 0.082) | 0.059 (0.043 ~ 0.075) | 0.055 (0.041 ~ 0.068) |
| 210 | 0.074 (0.056 ~ 0.092) | 0.063 (0.046 ~ 0.083) | 0.081 (0.062 ~ 0.100) | 0.077 (0.059 ~ 0.094) |
| 252 | 0.057 (0.043 ~ 0.072) | 0.066 (0.049 ~ 0.085) | 0.061 (0.044 ~ 0.079) | 0.044 (0.033 ~ 0.054) |
| 294 | 0.052 (0.040 ~ 0.065) | 0.061 (0.045 ~ 0.078) | 0.053 (0.040 ~ 0.065) | 0.043 (0.033 ~ 0.053) |
| 336 | 0.063 (0.047 ~ 0.079) | 0.065 (0.048 ~ 0.083) | 0.066 (0.049 ~ 0.082) | 0.058 (0.043 ~ 0.074) |
| 378 | 0.041 (0.029 ~ 0.053) | 0.046 (0.032 ~ 0.062) | 0.028 (0.020 ~ 0.036) | 0.048 (0.035 ~ 0.062) |
| 420 | 0.047 (0.035 ~ 0.059) | 0.045 (0.032 ~ 0.058) | 0.046 (0.035 ~ 0.058) | 0.050 (0.038 ~ 0.062) |
| 462 | 0.037 (0.028 ~ 0.047) | 0.056 (0.040 ~ 0.073) | 0.030 (0.021 ~ 0.038) | 0.027 (0.019 ~ 0.035) |
| 504 | 0.046 (0.034 ~ 0.058) | 0.028 (0.020 ~ 0.038) | 0.067 (0.050 ~ 0.085) | 0.043 (0.032 ~ 0.054) |
| 546 | 0.036 (0.027 ~ 0.046) | 0.041 (0.029 ~ 0.053) | 0.027 (0.019 ~ 0.035) | 0.041 (0.031 ~ 0.051) |
| 588 | 0.031 (0.023 ~ 0.040) | 0.032 (0.022 ~ 0.044) | 0.024 (0.017 ~ 0.032) | 0.037 (0.028 ~ 0.046) |
| 630 | 0.031 (0.023 ~ 0.041) | 0.031 (0.021 ~ 0.041) | 0.037 (0.027 ~ 0.048) | 0.026 (0.019 ~ 0.034) |
| 672 | 0.045 (0.034 ~ 0.056) | 0.041 (0.030 ~ 0.052) | 0.052 (0.040 ~ 0.065) | 0.042 (0.032 ~ 0.052) |
| 714 | 0.036 (0.027 ~ 0.044) | 0.045 (0.034 ~ 0.056) | 0.028 (0.021 ~ 0.036) | 0.033 (0.025 ~ 0.041) |
| 756 | 0.040 (0.030 ~ 0.051) | 0.054 (0.040 ~ 0.069) | 0.044 (0.033 ~ 0.056) | 0.023 (0.016 ~ 0.030) |
| 798 | 0.027 (0.020 ~ 0.033) | 0.035 (0.025 ~ 0.044) | 0.022 (0.016 ~ 0.027) | 0.024 (0.017 ~ 0.030) |
| 840 | 0.030 (0.023 ~ 0.037) | 0.045 (0.034 ~ 0.057) | 0.023 (0.017 ~ 0.029) | 0.020 (0.015 ~ 0.026) |
| 1260 | 0.024 (0.018 ~ 0.030) | 0.022 (0.016 ~ 0.028) | 0.023 (0.017 ~ 0.029) | 0.027 (0.020 ~ 0.034) |
| 1680 | 0.013 (0.009 ~ 0.017) | 0.017 (0.011 ~ 0.023) | 0.007 (0.004 ~ 0.011) | 0.014 (0.009 ~ 0.019) |
| 2100 | 0.023 (0.017 ~ 0.029) | 0.018 (0.013 ~ 0.023) | 0.013 (0.009 ~ 0.019) | 0.038 (0.029 ~ 0.047) |
| 2520 | 0.026 (0.020 ~ 0.033) | 0.025 (0.018 ~ 0.033) | 0.018 (0.013 ~ 0.024) | 0.036 (0.027 ~ 0.045) |
| 2940 | 0.019 (0.014 ~ 0.025) | 0.016 (0.010 ~ 0.021) | 0.014 (0.010 ~ 0.019) | 0.027 (0.020 ~ 0.034) |
| 3360 | 0.027 (0.020 ~ 0.034) | 0.035 (0.025 ~ 0.046) | 0.022 (0.017 ~ 0.029) | 0.022 (0.016 ~ 0.029) |
| 3780 | 0.021 (0.015 ~ 0.028) | 0.020 (0.013 ~ 0.028) | 0.013 (0.009 ~ 0.019) | 0.031 (0.023 ~ 0.040) |
| 4200 | 0.024 (0.018 ~ 0.030) | 0.017 (0.012 ~ 0.023) | 0.019 (0.013 ~ 0.025) | 0.036 (0.027 ~ 0.045) |
|  | B. Among Distill |  |  | C. Stratified-Random |
| N | GPT - Gemma | GPT - Qwen | Qwen - Gemma | Stratified-Random |
| 42 | -0.008 (-0.013 ~ -0.004) | 0.013 (0.008 ~ 0.018) | -0.021 (-0.027 ~ -0.015) | -0.005 (-0.018 ~ 0.010) |
| 84 | 0.001 (-0.003 ~ 0.006) | 0.003 (-0.002 ~ 0.008) | -0.002 (-0.007 ~ 0.003) | 0.007 (-0.003 ~ 0.019) |
| 126 | 0.014 (0.010 ~ 0.018) | 0.025 (0.018 ~ 0.032) | -0.011 (-0.016 ~ -0.007) | 0.027 (0.018 ~ 0.038) |
| 168 | 0.006 (0.003 ~ 0.010) | -0.004 (-0.010 ~ 0.001) | 0.011 (0.004 ~ 0.018) | 0.019 (0.012 ~ 0.026) |
| 210 | -0.018 (-0.027 ~ -0.008) | -0.004 (-0.009 ~ 0.000) | -0.013 (-0.021 ~ -0.005) | 0.011 (0.006 ~ 0.017) |
| 252 | 0.005 (0.000 ~ 0.010) | -0.018 (-0.028 ~ -0.009) | 0.023 (0.013 ~ 0.034) | 0.012 (0.007 ~ 0.017) |
| 294 | 0.009 (0.003 ~ 0.016) | -0.010 (-0.015 ~ -0.005) | 0.018 (0.009 ~ 0.029) | 0.015 (0.010 ~ 0.020) |
| 336 | -0.001 (-0.006 ~ 0.005) | -0.007 (-0.011 ~ -0.003) | 0.006 (0.001 ~ 0.013) | 0.014 (0.010 ~ 0.018) |
| 378 | 0.018 (0.009 ~ 0.028) | 0.020 (0.014 ~ 0.026) | -0.002 (-0.008 ~ 0.005) | 0.020 (0.014 ~ 0.026) |
| 420 | -0.002 (-0.006 ~ 0.003) | 0.004 (0.001 ~ 0.007) | -0.006 (-0.011 ~ 0.000) | 0.011 (0.008 ~ 0.014) |
| 462 | 0.026 (0.016 ~ 0.038) | -0.003 (-0.008 ~ 0.002) | 0.029 (0.016 ~ 0.044) | 0.021 (0.016 ~ 0.027) |
| 504 | -0.039 (-0.049 ~ -0.029) | -0.024 (-0.033 ~ -0.016) | -0.015 (-0.022 ~ -0.008) | 0.012 (0.008 ~ 0.016) |
| 546 | 0.014 (0.009 ~ 0.019) | 0.014 (0.009 ~ 0.019) | 0.000 (-0.005 ~ 0.006) | 0.010 (0.006 ~ 0.015) |
| 588 | 0.008 (0.002 ~ 0.015) | 0.013 (0.009 ~ 0.017) | -0.005 (-0.012 ~ 0.003) | 0.013 (0.008 ~ 0.019) |
| 630 | -0.007 (-0.011 ~ -0.002) | -0.011 (-0.015 ~ -0.006) | 0.004 (-0.001 ~ 0.009) | 0.011 (0.007 ~ 0.016) |
| 672 | -0.011 (-0.016 ~ -0.007) | -0.010 (-0.018 ~ -0.004) | -0.001 (-0.008 ~ 0.007) | 0.000 (-0.003 ~ 0.003) |
| 714 | 0.017 (0.011 ~ 0.022) | 0.005 (0.000 ~ 0.010) | 0.012 (0.006 ~ 0.018) | 0.004 (0.000 ~ 0.009) |
| 756 | 0.010 (0.004 ~ 0.017) | -0.021 (-0.029 ~ -0.014) | 0.031 (0.020 ~ 0.043) | 0.004 (0.001 ~ 0.007) |
| 798 | 0.013 (0.007 ~ 0.020) | 0.002 (-0.002 ~ 0.006) | 0.011 (0.003 ~ 0.019) | 0.004 (0.001 ~ 0.007) |
| 840 | 0.022 (0.015 ~ 0.030) | -0.003 (-0.006 ~ 0.000) | 0.025 (0.018 ~ 0.033) | 0.002 (0.000 ~ 0.006) |
| 1260 | -0.001 (-0.004 ~ 0.003) | 0.004 (-0.001 ~ 0.009) | -0.005 (-0.010 ~ 0.001) | -0.005 (-0.008 ~ -0.002) |
| 1680 | 0.010 (0.005 ~ 0.014) | 0.007 (0.002 ~ 0.012) | 0.003 (-0.002 ~ 0.009) | 0.000 (-0.002 ~ 0.002) |
| 2100 | 0.005 (0.001 ~ 0.008) | 0.025 (0.018 ~ 0.032) | -0.020 (-0.026 ~ -0.015) | -0.001 (-0.003 ~ 0.001) |
| 2520 | 0.006 (0.002 ~ 0.011) | 0.017 (0.012 ~ 0.023) | -0.011 (-0.017 ~ -0.005) | -0.006 (-0.008 ~ -0.004) |
| 2940 | 0.001 (-0.002 ~ 0.005) | 0.013 (0.009 ~ 0.017) | -0.012 (-0.016 ~ -0.007) | 0.001 (0.000 ~ 0.002) |
| 3360 | 0.013 (0.007 ~ 0.020) | 0.000 (-0.003 ~ 0.003) | 0.013 (0.006 ~ 0.021) | 0.001 (-0.001 ~ 0.002) |
| 3780 | 0.006 (0.002 ~ 0.011) | 0.018 (0.013 ~ 0.023) | -0.011 (-0.016 ~ -0.007) | -0.003 (-0.005 ~ -0.002) |
| 4200 | -0.002 (-0.005 ~ 0.002) | 0.017 (0.012 ~ 0.023) | -0.019 (-0.025 ~ -0.013) | 0.000 (0.000 ~ 0.001) |
|  | D. Student - teacher |  |  | E. Human - LLM |
| N | Gemma | GPT | Qwen | Human - Gemma |
| 42 | -0.042 (-0.059 ~ -0.027) | -0.067 (-0.090 ~ -0.048) | -0.056 (-0.076 ~ -0.038) | -0.043 (-0.057 ~ -0.030) |
| 84 | -0.063 (-0.081 ~ -0.045) | -0.079 (-0.098 ~ -0.060) | -0.058 (-0.076 ~ -0.040) | -0.030 (-0.043 ~ -0.016) |
| 126 | -0.073 (-0.094 ~ -0.052) | -0.076 (-0.098 ~ -0.056) | -0.077 (-0.100 ~ -0.057) | -0.016 (-0.028 ~ -0.004) |
| 168 | -0.072 (-0.092 ~ -0.051) | -0.083 (-0.105 ~ -0.061) | -0.054 (-0.071 ~ -0.039) | 0.003 (-0.008 ~ 0.015) |
| 210 | -0.062 (-0.083 ~ -0.042) | -0.097 (-0.119 ~ -0.074) | -0.068 (-0.087 ~ -0.050) | 0.007 (-0.003 ~ 0.019) |
| 252 | -0.066 (-0.085 ~ -0.046) | -0.077 (-0.099 ~ -0.057) | -0.036 (-0.048 ~ -0.024) | 0.007 (-0.004 ~ 0.018) |
| 294 | -0.061 (-0.080 ~ -0.042) | -0.069 (-0.087 ~ -0.052) | -0.035 (-0.048 ~ -0.023) | 0.008 (-0.003 ~ 0.020) |
| 336 | -0.058 (-0.077 ~ -0.041) | -0.076 (-0.096 ~ -0.057) | -0.045 (-0.062 ~ -0.029) | 0.013 (0.003 ~ 0.025) |
| 378 | -0.047 (-0.065 ~ -0.030) | -0.046 (-0.059 ~ -0.033) | -0.042 (-0.059 ~ -0.026) | 0.010 (-0.001 ~ 0.022) |
| 420 | -0.037 (-0.052 ~ -0.022) | -0.056 (-0.071 ~ -0.042) | -0.036 (-0.049 ~ -0.024) | 0.013 (0.003 ~ 0.025) |
| 462 | -0.056 (-0.075 ~ -0.038) | -0.047 (-0.059 ~ -0.034) | -0.020 (-0.030 ~ -0.010) | 0.011 (0.000 ~ 0.022) |
| 504 | -0.019 (-0.031 ~ -0.007) | -0.075 (-0.095 ~ -0.055) | -0.027 (-0.039 ~ -0.016) | 0.015 (0.005 ~ 0.027) |
| 546 | -0.035 (-0.049 ~ -0.021) | -0.038 (-0.050 ~ -0.026) | -0.028 (-0.041 ~ -0.016) | 0.012 (0.002 ~ 0.023) |
| 588 | -0.023 (-0.037 ~ -0.011) | -0.033 (-0.043 ~ -0.022) | -0.021 (-0.033 ~ -0.010) | 0.016 (0.005 ~ 0.027) |
| 630 | -0.022 (-0.035 ~ -0.010) | -0.046 (-0.059 ~ -0.033) | -0.011 (-0.023 ~ 0.000) | 0.014 (0.004 ~ 0.026) |
| 672 | -0.025 (-0.037 ~ -0.014) | -0.054 (-0.068 ~ -0.040) | -0.019 (-0.029 ~ -0.010) | 0.016 (0.005 ~ 0.027) |
| 714 | -0.029 (-0.041 ~ -0.017) | -0.029 (-0.039 ~ -0.020) | -0.010 (-0.020 ~ -0.001) | 0.018 (0.008 ~ 0.030) |
| 756 | -0.036 (-0.049 ~ -0.023) | -0.043 (-0.056 ~ -0.031) | 0.002 (-0.008 ~ 0.011) | 0.020 (0.009 ~ 0.032) |
| 798 | -0.017 (-0.027 ~ -0.007) | -0.021 (-0.029 ~ -0.013) | 0.001 (-0.008 ~ 0.010) | 0.020 (0.009 ~ 0.031) |
| 840 | -0.024 (-0.035 ~ -0.013) | -0.019 (-0.026 ~ -0.011) | 0.008 (-0.003 ~ 0.019) | 0.023 (0.012 ~ 0.035) |
| 1260 | 0.001 (-0.008 ~ 0.011) | -0.017 (-0.024 ~ -0.010) | 0.003 (-0.005 ~ 0.011) | 0.021 (0.010 ~ 0.032) |
| 1680 | 0.007 (-0.002 ~ 0.018) | 0.000 (-0.007 ~ 0.006) | 0.017 (0.008 ~ 0.026) | 0.024 (0.013 ~ 0.037) |
| 2100 | 0.009 (-0.002 ~ 0.020) | -0.004 (-0.010 ~ 0.002) | -0.005 (-0.014 ~ 0.004) | 0.026 (0.015 ~ 0.038) |
| 2520 | 0.005 (-0.005 ~ 0.015) | -0.006 (-0.012 ~ 0.000) | 0.001 (-0.008 ~ 0.010) | 0.027 (0.015 ~ 0.039) |
| 2940 | 0.014 (0.003 ~ 0.025) | -0.002 (-0.008 ~ 0.003) | 0.009 (0.000 ~ 0.017) | 0.029 (0.018 ~ 0.042) |
| 3360 | -0.007 (-0.017 ~ 0.004) | -0.011 (-0.017 ~ -0.005) | 0.013 (0.005 ~ 0.023) | 0.029 (0.018 ~ 0.042) |
| 3780 | 0.009 (-0.001 ~ 0.019) | -0.002 (-0.008 ~ 0.004) | 0.004 (-0.006 ~ 0.014) | 0.027 (0.015 ~ 0.039) |
| 4200 | 0.008 (-0.001 ~ 0.019) | -0.011 (-0.018 ~ -0.003) | -0.004 (-0.013 ~ 0.005) | 0.026 (0.015 ~ 0.038) |
|  | E. Human - LLM |  | G. Recall of Negation subtypes | |
| N | Human - GPT | Human - Qwen | Sentential | Telegraphic |
| 42 | -0.060 (-0.076 ~ -0.046) | -0.036 (-0.051 ~ -0.023) | -0.013 (-0.019 ~ -0.008) | 0.408 (0.350 ~ 0.465) |
| 84 | -0.047 (-0.058 ~ -0.036) | -0.023 (-0.034 ~ -0.012) | -0.005 (-0.008 ~ -0.002) | 0.310 (0.264 ~ 0.355) |
| 126 | -0.034 (-0.044 ~ -0.024) | -0.010 (-0.021 ~ 0.002) | 0.001 (-0.001 ~ 0.002) | 0.328 (0.294 ~ 0.365) |
| 168 | -0.014 (-0.022 ~ -0.006) | 0.010 (-0.001 ~ 0.021) | -0.002 (-0.004 ~ 0.000) | 0.139 (0.114 ~ 0.163) |
| 210 | -0.010 (-0.017 ~ -0.003) | 0.014 (0.003 ~ 0.025) | 0.001 (0.000 ~ 0.004) | 0.147 (0.117 ~ 0.174) |
| 252 | -0.010 (-0.017 ~ -0.003) | 0.014 (0.003 ~ 0.025) | -0.004 (-0.006 ~ -0.001) | 0.077 (0.049 ~ 0.107) |
| 294 | -0.009 (-0.016 ~ -0.002) | 0.015 (0.004 ~ 0.027) | -0.003 (-0.006 ~ -0.001) | 0.074 (0.042 ~ 0.110) |
| 336 | -0.004 (-0.010 ~ 0.003) | 0.020 (0.009 ~ 0.032) | -0.003 (-0.005 ~ -0.001) | 0.040 (0.020 ~ 0.063) |
| 378 | -0.007 (-0.014 ~ -0.001) | 0.017 (0.005 ~ 0.028) | -0.003 (-0.005 ~ -0.001) | 0.067 (0.033 ~ 0.102) |
| 420 | -0.004 (-0.010 ~ 0.003) | 0.020 (0.009 ~ 0.031) | 0.000 (-0.002 ~ 0.002) | 0.054 (0.030 ~ 0.081) |
| 462 | -0.007 (-0.013 ~ 0.000) | 0.018 (0.007 ~ 0.029) | -0.001 (-0.003 ~ 0.001) | 0.043 (0.017 ~ 0.068) |
| 504 | -0.002 (-0.008 ~ 0.005) | 0.022 (0.011 ~ 0.034) | 0.001 (0.000 ~ 0.002) | 0.066 (0.039 ~ 0.095) |
| 546 | -0.006 (-0.012 ~ 0.001) | 0.018 (0.008 ~ 0.030) | 0.001 (-0.001 ~ 0.002) | 0.069 (0.033 ~ 0.108) |
| 588 | -0.001 (-0.008 ~ 0.006) | 0.023 (0.011 ~ 0.034) | 0.001 (0.000 ~ 0.003) | 0.079 (0.041 ~ 0.119) |
| 630 | -0.003 (-0.010 ~ 0.004) | 0.021 (0.010 ~ 0.033) | -0.001 (-0.003 ~ 0.001) | 0.050 (0.014 ~ 0.091) |
| 672 | -0.001 (-0.008 ~ 0.006) | 0.023 (0.012 ~ 0.034) | -0.001 (-0.002 ~ 0.000) | 0.015 (-0.015 ~ 0.047) |
| 714 | 0.001 (-0.005 ~ 0.008) | 0.025 (0.014 ~ 0.037) | 0.001 (-0.001 ~ 0.004) | 0.042 (0.007 ~ 0.081) |
| 756 | 0.003 (-0.003 ~ 0.010) | 0.027 (0.016 ~ 0.039) | -0.001 (-0.002 ~ 0.000) | 0.036 (0.019 ~ 0.053) |
| 798 | 0.003 (-0.004 ~ 0.010) | 0.027 (0.015 ~ 0.038) | -0.001 (-0.003 ~ 0.001) | 0.013 (-0.013 ~ 0.042) |
| 840 | 0.006 (-0.001 ~ 0.013) | 0.030 (0.018 ~ 0.042) | 0.000 (-0.001 ~ 0.001) | 0.035 (0.001 ~ 0.072) |
| 1260 | 0.003 (-0.003 ~ 0.010) | 0.027 (0.016 ~ 0.039) | -0.002 (-0.004 ~ -0.001) | -0.001 (-0.016 ~ 0.017) |
| 1680 | 0.007 (0.001 ~ 0.014) | 0.031 (0.019 ~ 0.043) | 0.001 (0.000 ~ 0.002) | 0.018 (0.009 ~ 0.028) |
| 2100 | 0.009 (0.002 ~ 0.016) | 0.033 (0.021 ~ 0.045) | -0.001 (-0.002 ~ 0.000) | 0.005 (-0.012 ~ 0.021) |
| 2520 | 0.010 (0.003 ~ 0.017) | 0.034 (0.022 ~ 0.046) | 0.000 (-0.002 ~ 0.001) | -0.011 (-0.024 ~ 0.001) |
| 2940 | 0.012 (0.006 ~ 0.020) | 0.036 (0.024 ~ 0.049) | 0.000 (0.000 ~ 0.000) | 0.002 (-0.004 ~ 0.007) |
| 3360 | 0.012 (0.005 ~ 0.019) | 0.036 (0.024 ~ 0.048) | 0.000 (0.000 ~ 0.001) | 0.013 (0.004 ~ 0.023) |
| 3780 | 0.010 (0.003 ~ 0.017) | 0.034 (0.022 ~ 0.046) | 0.000 (-0.001 ~ 0.001) | -0.003 (-0.010 ~ 0.003) |
| 4200 | 0.008 (0.002 ~ 0.016) | 0.032 (0.021 ~ 0.044) | -0.001 (-0.001 ~ 0.000) | -0.001 (-0.003 ~ 0.001) |
|  | G. Recall of Negation subtypes | |  |  |
| N | Pseudo-speculation | Morphological |  |  |
| 42 | 0.348 (0.065 ~ 0.704) | N/A |  |  |
| 84 | 0.344 (0.143 ~ 0.611) | N/A |  |  |
| 126 | 0.091 (-0.254 ~ 0.537) | N/A |  |  |
| 168 | 0.336 (0.074 ~ 0.694) | N/A |  |  |
| 210 | 0.094 (-0.167 ~ 0.389) | N/A |  |  |
| 252 | 0.176 (-0.111 ~ 0.556) | N/A |  |  |
| 294 | 0.191 (-0.051 ~ 0.540) | N/A |  |  |
| 336 | 0.299 (0.086 ~ 0.481) | N/A |  |  |
| 378 | 0.187 (-0.056 ~ 0.519) | N/A |  |  |
| 420 | 0.112 (-0.130 ~ 0.444) | N/A |  |  |
| 462 | 0.081 (-0.063 ~ 0.278) | N/A |  |  |
| 504 | 0.113 (0.037 ~ 0.204) | N/A |  |  |
| 546 | 0.295 (0.167 ~ 0.426) | N/A |  |  |
| 588 | 0.237 (0.056 ~ 0.394) | N/A |  |  |
| 630 | 0.102 (0.000 ~ 0.238) | N/A |  |  |
| 672 | -0.133 (-0.278 ~ 0.022) | N/A |  |  |
| 714 | 0.211 (0.100 ~ 0.333) | N/A |  |  |
| 756 | -0.115 (-0.315 ~ 0.095) | N/A |  |  |
| 798 | -0.252 (-0.574 ~ 0.086) | N/A |  |  |
| 840 | 0.026 (-0.056 ~ 0.097) | N/A |  |  |
| 1260 | -0.184 (-0.467 ~ 0.093) | N/A |  |  |
| 1680 | 0.018 (-0.071 ~ 0.139) | N/A |  |  |
| 2100 | -0.062 (-0.111 ~ -0.012) | N/A |  |  |
| 2520 | -0.140 (-0.278 ~ -0.037) | N/A |  |  |
| 2940 | 0.013 (-0.063 ~ 0.083) | N/A |  |  |
| 3360 | 0.013 (-0.063 ~ 0.083) | N/A |  |  |
| 3780 | 0.015 (-0.028 ~ 0.067) | N/A |  |  |
| 4200 | 0.036 (0.000 ~ 0.067) | N/A |  |  |

Table S1.	Mean differences (ΔF1) with 95% percentile bootstrap confidence

intervals are reported for each training data size (N). For group

comparisons, performance within each group was averaged across all

eligible model configurations (backbone architecture, label source,

sampling strategy, and data seeds as applicable) within each bootstrap

replicate prior to computing paired differences. Positive values indicate

higher mean F1 scores for the first setting relative to the second.

As these analyses were considered secondary and descriptive, we did

not conduct formal hypothesis testing at the individual N level.

A. Human–Distill through G. Recall of Negation Subtypes, including all

corresponding subcomponents, follow the definitions specified in Table

6 (Primary Comparative Analysis). GPT: GPT-5.3 Chat; Qwen: Qwen3.6-

Plus; Gemma: Gemma 4. Morphological negation did not appear in the

test dataset(n = 0), precluding the calculation of recall.
