# Supplementary material for: Reducing Annotation Costs Using Knowledge Distillation for Label‑Efficient Negation Detection in Radiology Reports: Multisite Internal Validation Study
Source: JMIR Med Inform. 2026 Oct 22;14:e103912. doi: 10.2196/103912 (PMC13740815; doi:10.2196/103912)
Supplement: Supplementary file 4 [file medinform_v14i1e103912_app4.docx]

**Figure S1.**	Per-N secondary analysis of Comparison Families A and B. Zoomed: zoomed views

with the y-axis starting at F1 = 0.75.

**Figure S2.**	Per-N secondary analysis of Comparison Families C and D. Zoomed: zoomed views

with the y-axis starting at F1 = 0.75.

**Figure S3.**	Per-N secondary analysis of Comparison Families E and G. Zoomed: zoomed views

with the y-axis starting at F1 = 0.75. Sentential: Sentential negation;

Telegraphic: Symbolic or telegraphic-style negation; Pseudo-speculation: Speculation

unrelated to abnormal findings; Morphological negation was absent in the test dataset

(n = 0), precluding recall calculation.
